# Association of Unilateral Radiotherapy With Contralateral Lymph Node Failure Among Patients With Squamous Cell Carcinoma of the Tonsil: A Systematic Review and Meta-analysis

**DOI:** 10.1001/jamanetworkopen.2022.55209

**Published:** 2023-02-08

**Authors:** Niema B. Razavian, Ralph B. D’Agostino, Cole R. Steber, Corbin A. Helis, Ryan T. Hughes

**Affiliations:** 1 Department of Radiation Oncology, Wake Forest University School of Medicine, Winston-Salem, North Carolina; 2 Department of Biostatistics and Data Science, Wake Forest University School of Medicine, Winston-Salem, North Carolina; 3 Department of Radiation Oncology, Fort Belvoir Community Hospital, Fort Belvoir, Virginia

## Abstract

**Importance:**

Ipsilateral neck radiotherapy (RT) is controversial in some patients with tonsil cancer due to concern for nodal failure within the contralateral nonirradiated neck (hereinafter referred to as contralateral neck failure [CNF]).

**Objective:**

To determine the rate of CNF following ipsilateral neck RT in patients with tonsil cancer.

**Data Sources:**

Databases including PubMed, Embase, Web of Science, and Cochrane Library were queried for peer-reviewed, English language articles published between January 1, 1980, and December 31, 2021.

**Study Selection:**

Studies reporting rates of CNF from at least 20 patients treated with ipsilateral neck RT. Studies were excluded if they lacked full text, reported results from databases or systematic reviews, or did not provide RT details.

**Data Extraction and Synthesis:**

Data were extracted following the PRISMA reporting guideline. Study quality was assessed using criteria from a methodological index for nonrandomized studies. Pooled outcomes were estimated using random-effects models.

**Main Outcomes and Measures:**

Primary outcome was the pooled rate of CNF following ipsilateral neck RT. Secondary outcomes were the pooled rates of CNF by tumor and nodal staging categories from the 7th edition of the *AJCC Cancer Staging Manual* and rates of toxic effects.

**Results:**

A total of 17 studies (16 retrospective and 1 prospective) including 1487 unique patients were identified. The pooled risk of CNF was 1.9% (95% CI, 1.2%-2.6%). The rate of CNF by tumor (T) category was as follows: 1.3% (95% CI, 0.3%-2.3%) for T1; 3.0% (95% CI, 1.6%-4.4%) for T2; 11.3% (95% CI, 3.3%-19.2%) for T3; and 16.0% (95% CI, −7.8% to 39.8%) for T4. Patients with T3 to T4 tumors had a significantly higher rate of CNF than those with T1 to T2 tumors (11.5% [95% CI, 3.9%-19.1%] vs 1.8% [95% CI, 1.0%-2.6%]; *P* < .001). The rate of CNF by nodal (N) category was 1.2% (95% CI, 0.1%-2.2%) for N0; 4.8% (95% CI, 2.4%-7.2%) for N1; 3.1% (95% CI, 0.4%-5.8%) for N2a; 3.1% (95% CI, 1.2%-4.9%) for N2b; and 0 (95% CI, not applicable) for N3. Rates of CNF were similar for patients with N2b to N3 and N0 to N2a disease (3.0% [95% CI, 1.2%-4.7%] vs 1.7% [95% CI, 0.6%-2.8%], respectively; *P* = .07). Compared with bilateral RT, ipsilateral RT was associated with increased risk of CNF (log odds ratio, 1.29 [95% CI, 0.09-2.48]; *P* = .04). The crude rates of xerostomia of grade 3 or greater and feeding tube use were 0.9% (95% CI, –0.2% to 1.9%) and 13.3% (95% CI, 8.3%-18.3%), respectively.

**Conclusions and Relevance:**

In this systematic review and meta-analysis, ipsilateral neck RT was associated with a low rate of CNF in patients with small, lateralized tonsil cancers. Bilateral neck RT was associated with lower risk of CNF compared with ipsilateral neck RT. Patients with tumors of a higher T category were at increased risk for CNF following ipsilateral neck RT, and advanced nodal stage was not associated with CNF. Rates of toxic effects appeared favorable in patients treated with ipsilateral neck RT.

## Key Points

**Question:**

What is the risk of lymph node failure within the contralateral nonirradiated neck when irradiating only the ipsilateral neck in patients with tonsil cancer?

**Findings:**

In this systematic review and meta-analysis of 17 studies with 1487 unique patients with tonsil cancer who received ipsilateral neck radiotherapy, the rate of contralateral nodal failure was 2%.

**Meaning:**

These findings suggest that ipsilateral neck radiotherapy is associated with a low risk of contralateral nodal failure and should be considered in patients with small, lateralized tonsil cancers.

## Teaser

This systematic review and meta-analysis assesses the risk of nodal failure within the contralateral nonirradiated neck after ipsilateral neck radiotherapy in patients with tonsil cancer.

## Introduction

Radiotherapy (RT) is an established treatment paradigm for patients with early-stage and locally advanced squamous cell carcinoma (SCC) of the tonsil in both the definitive and postoperative settings.^1,2,3,4,5^ Historically, RT fields targeted both the primary tumor and bilateral neck, even when the contralateral neck was clinically (or pathologically) negative. Omission of the uninvolved, contralateral neck from the RT target is controversial, particularly for patients with more than 1 involved lymph node.^6^ There is significant morbidity attributed to bilateral neck irradiation, and safe omission of RT to the contralateral neck could greatly improve acute and late toxic effects as well as patient quality of life.^7,8,9^ To adequately personalize RT targets to each patient’s individualized estimated risk of nodal failure within the contralateral nonirradiated neck (contralateral neck failure [CNF]) based on clinical and pathologic factors, further high-quality evidence is required.

Single-institution, retrospective studies of ipsilateral neck RT for oropharyngeal cancer^10,11,12,13^ have demonstrated mixed results with regard to CNF, with rates ranging from 0% to 13%. Prospective studies on this topic are limited, with CNF rates reported to range from 0% to 2.7%.^14,15^ There have been no randomized clinical trials examining the utility of ipsilateral vs bilateral RT in patients with lateralized tonsil cancer, likely due to issues of equipoise, logistics, patient preferences, and prohibitive costs. As a result, currently available clinical practice guidelines on this topic are entirely based on level 2 to 3 evidence, and expert consensus recommendations are heterogeneous with low to moderate strength of evidence. Current guidelines recommend ipsilateral neck RT only in patients with well-lateralized primary tumors and a single involved lymph node, while bilateral neck RT is recommended for patients with involvement of more than 1 ipsilateral lymph node or any contralateral lymph nodes.^16,17,18^ A clear understanding of the risk of CNF and of factors associated with CNF after ipsilateral neck RT for tonsil cancer would strongly inform clinical practice in an area with overall weak supporting evidence.

We performed a systematic review and meta-analysis to better understand the risk of CNF after ipsilateral neck RT for patients with tonsil cancer. The primary objective of this study was to determine the rate of CNF following ipsilateral neck RT. The association of prognostic factors such as tumor (T) category and nodal (N) category on CNF were also examined. Additionally, RT-related morbidity was assessed. An improved understanding of this treatment modality will allow for more comprehensive risk-benefit discussions as well as provide preliminary data to inform clinical trials in areas where the optimal balance of risk and benefit is currently unclear.

## Methods

We performed a systematic literature review and meta-analysis following the Preferred Reporting Items for Systematic Reviews and Meta-analyses (PRISMA) reporting guideline.^19^ Review and analysis were designed prospectively and registered with PROSPERO (CRD42021237637). In total, 4 databases (PubMed, Embase, Web of Science, and Cochrane Library) were queried using standardized search terms (listed in the eMethods in Supplement 1) to identify publications that included the terms *tonsil cancer*, *ipsilateral radiation*, and *contralateral neck failure*. Only peer-reviewed articles published between January 1, 1980, and December 31, 2021, were included for screening. After removing duplicate publications, titles and abstracts were screened by at least 2 reviewers (including N.B.R., C.R.S., and C.A.H.). In the case of discordance, a third reviewer (R.T.H.) was added, and inclusion for full text review was determined by consensus opinion. To reduce bias from small patient populations, articles were included for analysis if they contained at least 20 patients treated with ipsilateral neck RT for tonsil cancer. Additionally, in studies with overlapping patient populations, only the largest study was included for analysis to minimize confounding. A full list of inclusion and exclusion criteria (and the number of corresponding articles) is provided in the eMethods in Supplement 1. Following full text review, rates of CNF failure, xerostomia of grade 3 or greater, and feeding tube use were recorded. Patient and treatment characteristics—such as RT modality, *AJCC Cancer Staging Manual* (*AJCC*) edition, T category, N category, use of surgery, chemotherapy use, smoking status, and human papillomavirus (HPV) status—were also extracted. Quality of studies included for statistical analysis was quantified using criteria from a methodological index for nonrandomized studies (MINORS).^20^ Publication bias was assessed using the Egger regression test for funnel plot symmetry.

The primary outcome was the pooled rate of CNF following ipsilateral neck RT. We defined CNF as a nodal failure within the contralateral neck that did not receive RT, as determined by clinical, pathologic, or radiographic assessments (eTable 1 in Supplement 1). Pooled rates of CNF were estimated using a random-effects model. Heterogeneity among the studies was assessed using Cochran *Q* tests for heterogeneity and the Higgins *I^2^* statistic. In cases of high heterogeneity (*I^2^* > 50% or *P* value of Cochran *Q* < .05), outlier studies were identified using Cook distance, and pooled rates were recalculated with the outlier studies removed. Sensitivity analyses to assess the association of tumor laterality, diagnostic imaging, and *AJCC* edition with CNF were performed. Differences between subgroups were evaluated using the omnibus test of moderators (Q_M_ statistic). Univariate meta-regression analyses were performed to examine the association between CNF and RT modality, HPV status, smoking status, use of surgery, and use of chemotherapy. To examine the association of CNF with dichotomous variables (eg, ipsilateral RT vs bilateral RT), mixed-effects meta-analyses comparing log odds ratios (ORs) across studies were performed. For these analyses, only studies that reported CNF in patients treated with each variable (eg, CNF after ipsilateral RT vs CNF after bilateral RT) were included. Statistical significance was defined as 2-sided *P* < .05. Detailed explanations of each analysis are presented in the eMethods in Supplement 1. All tests were performed using the metafor package, version 3.0-2, in R Studio, version 1.3.1073 2009-2020 (R Program for Statistical Computing).^21^

## Results

### Characteristics of Included Studies

**Figure 1.**
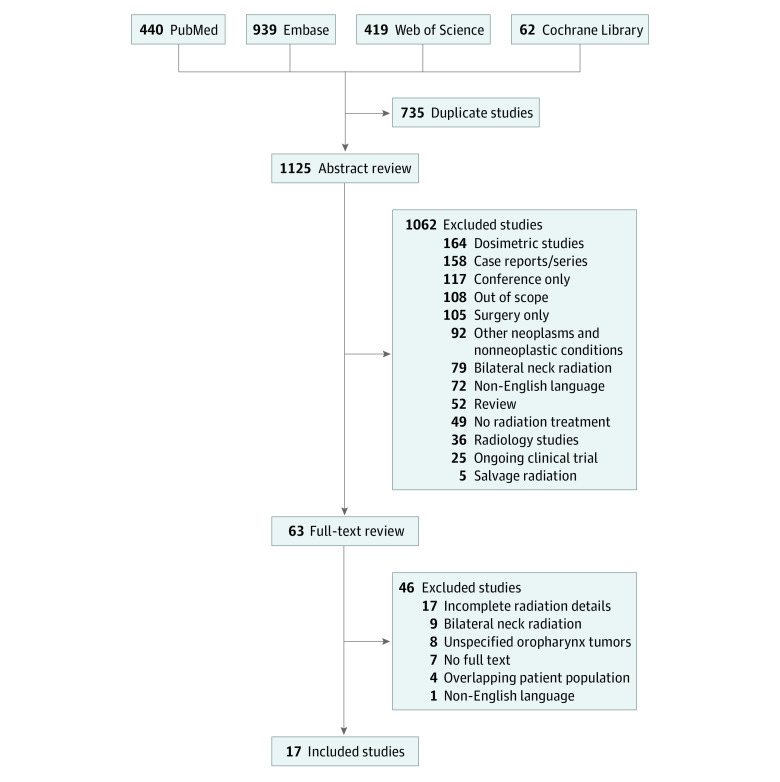
Study Flowchart

After removing duplicate citations, 1125 abstracts were screened, and 63 articles underwent full text review (Figure 1). In total, 17 studies (1487 unique patients) were included for statistical analysis.^10,11,15,22,23,24,25,26,27,28,29,30,31,32,33,34,35^ Baseline characteristics, treatments, and outcomes from the included studies are summarized in eTables 1 to 6 in Supplement 1. Included studies were published between 1980 and 2019. Most studies were retrospective (16 of 17 studies^10,11,22,23,24,25,26,27,28,29,30,31,32,33,34,35^), and the median follow-up ranged from 32.0 to 99.6 months. The median study quality by MINORS criteria was 10 (eTable 7 in Supplement 1), and publication bias was not identified by the Egger regression test (*P* = .15) (eFigure 1 in Supplement 1). Radiotherapy was designed using either 3-dimensional conformal RT (11 studies^10,11,15,24,26,28,29,30,31,32,35^) or intensity-modulated RT (IMRT; 11 studies^11,15,22,23,24,25,26,27,28,29,31,35^) and delivered in either the definitive (13 studies^11,15,22,24,25,26,27,28,30,32,34,35^) or postoperative (10 studies^10,11,23,25,26,27,28,29,31,32,35^) setting. The *AJCC* 7th edition (*AJCC*-7) was the most frequently used system for tumor and nodal staging. Human papillomavirus status was reported in 11 studies^10,15,23,24,25,26,28,29,30,32,35^; smoking history, in 11 studies^10,11,23,24,25,26,29,30,31,32,35^; and use of chemotherapy, in 13 studies.^10,11,15,23,24,25,26,27,28,29,31,32,35^

### Risk of CNF After Ipsilateral Neck RT

**Figure 2.**
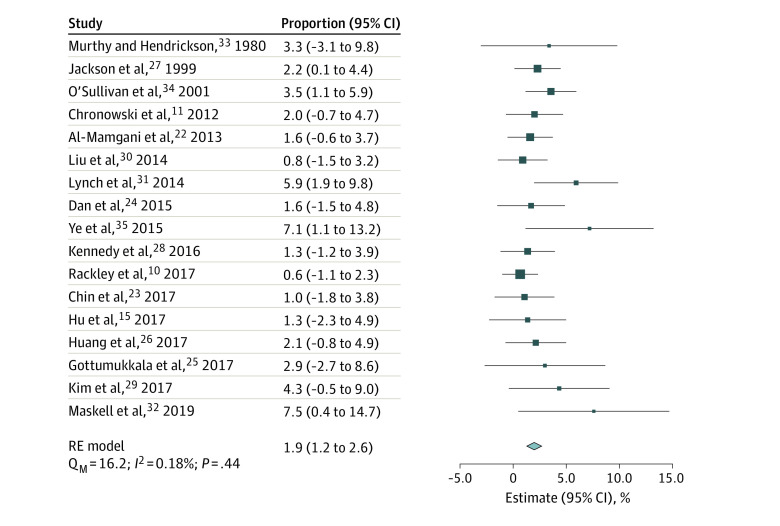
Forest Plot and Pooled Estimates of Contralateral Neck Failure Following Ipsilateral Neck Radiotherapy for All Included Studies Size of markers indicates relative sample size of study; diamond, heterogeneity; Q_M_, omnibus test of moderators; RE, random-effects.

From 1487 patients treated with ipsilateral neck RT, the pooled rate of CNF was 1.9% (95% CI, 1.2%-2.6%; *I^2^* = 0.18%) (Figure 2). After removal of lower-quality studies (n = 2), the pooled rate of CNF remained 1.9% (95% CI, 1.1%-2.7%; *I^2^* = 3.6%) (eFigure 2 in Supplement 1). When analyzed by staging system, CNF rate did not differ significantly between studies that used *AJCC*-7 (n = 545) (2.3% [95% CI, 1.0%-3.5%]; *I^2^* = 0.9%), that used an earlier edition (*AJCC*-1 through *AJCC*-5) (n = 494) (2.2% [95% CI, 0.9%-3.6%]; *I^2^* = 5.6%), or that did not provide a staging edition (n = 448) (1.5% [95% CI, 0.4%-2.6%]; *I^2^* = 2.7%) (Q_M_ test, *P* = .57) (eFigure 3 in Supplement 1). Additionally, the use of diagnostic imaging was not associated with a lower rate of CNF: the CNF rate in studies that used diagnostic imaging (1.5% [95% CI, 0.5%-2.5%]; *I^2^* = 0.1%) and those that did not use diagnostic imaging (2.3% [95% CI, 1.3%-3.3%]; *I^2^* = 0%) were not significantly different (Q_M_ test, *P* = .27) (eFigure 4 in Supplement 1).

### Association of T and N Stage With CNF

**Figure 3.**
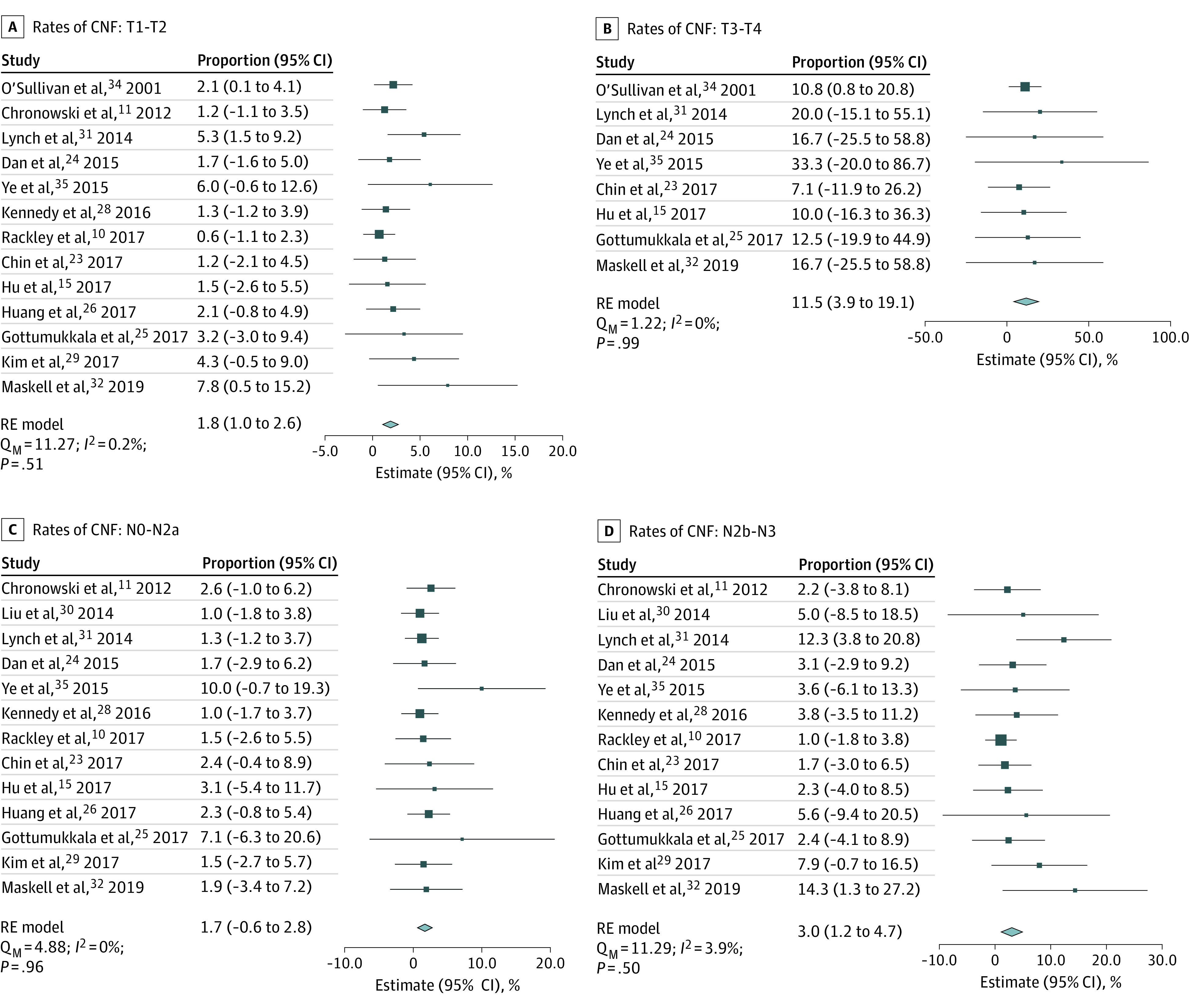
Forest Plot and Pooled Estimates of Contralateral Neck Failure (CNF) by T and N Stage Size of markers indicates relative sample size of study; diamond, heterogeneity; Q_M_, omnibus test of moderators; RE, random-effects.

The association of T and N category with ipsilateral neck RT outcomes was also examined. Across the included studies, there were 458 patients with T1, 537 with T2, 54 with T3, and 8 with T4 primary tumors. The rate of CNF following ipsilateral neck RT increased by T category: 1.3% (95% CI, 0.3%-2.3%; *I^2^* = 0.7%) for T1, 3.0% (95% CI, 1.6%-4.4%; *I^2^* = 0%) for T2, 11.3% (95% CI, 3.3%-19.2%; *I^2^* = 0%) for T3, and 16.0% (95% CI, −7.8% to 39.8%; *I^2^* = 0%) for T4 primary tumors (eFigure 5 in Supplement 1). The rate of CNF after ipsilateral neck RT was significantly greater (log OR, 1.65 [95% CI, 0.74-2.56]; *P* < .001) (eFigure 6 in Supplement 1) for T3 to T4 tumors (11.5% [95% CI, 3.9%-19.1%]; *I^2^* = 0%) than for T1 to T2 tumors (1.8% [95% CI, 1.0%-2.6%]; *I^2^* = 0.2%) (Figure 3A and B). However, the degree of primary tumor extension toward midline was not associated with significant differences in CNF rates among those with no extension (1.5% [95% CI, 0.7%-2.3%]), some extension (2.6% [95% CI, 0.1%-5.2%]), or extension not provided (4.0% [95% CI, 2.1%-5.9%]; Q_M_ test, *P* = .06) (eFigure 7 in Supplement 1).

Among the included studies, 474 patients with N0, 295 with NI, 146 with N2a, 339 with N2b, and 32 with N3 disease were available for subgroup analysis. Following ipsilateral neck RT, the pooled rates of CNF were 1.2% (95% CI, 0.1%-2.2%; *I^2^* = 6.3%) for N0, 4.8% (95% CI, 2.4%-7.2%; *I^2^* = 0%) for N1, 3.1% (95% CI, 0.4%-5.8%; *I^2^* = 0%) for N2a, 3.1% (95% CI, 1.2%-4.9%; *I^2^* = 5.8%) for N2b, and 0% (95% CI, not applicable; *I^2^* = not applicable) for N3 (eFigure 8 in Supplement 1) disease. Rates of CNF after ipsilateral neck RT were similar (log OR, 0.81 [95% CI −0.07 to 1.68]; *P* = .07) (eFigure 8 in Supplement 1) for N0 to N2a (1.7% [95% CI, 0.6%-2.8%]; *I^2^* = 0%) and N2b to N3 (3.0% [95% CI, 1.2%-4.7%]; *I^2^* = 3.9%) disease (Figure 3C and D).

### Comparison of Ipsilateral vs Bilateral Neck RT

**Figure 4.**
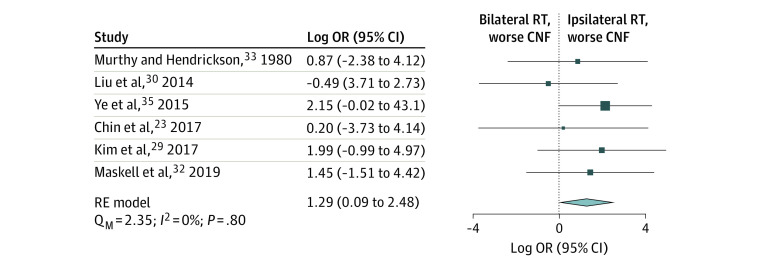
Forest Plot and Pooled Estimate of Log Odds Ratio (OR) of Contralateral Neck Failure for Comparison of Ipsilateral vs Bilateral Neck Radiotherapy Size of markers indicates relative sample size of study; diamond, heterogeneity; Q_M_, omnibus test of moderators; RE, random-effects.

In addition to reporting outcomes after ipsilateral neck RT, 6 studies^23,29,30,32,33,35^ reported CNF rates following bilateral neck RT. Among these 6 studies, 329 patients received ipsilateral neck RT and 395 patients received bilateral neck RT. The rate of CNF following ipsilateral neck RT estimated from the meta-analysis was 2.8% (95% CI, 0.6%-5.0%; *I^2^* = 38%) (eFigure 10, top, in Supplement 1), while the rate of CNF following bilateral neck RT was 0.9% (95% CI, 0-1.8%; *I^2^* = 0%) (eFigure 10, bottom, in Supplement 1). Compared with bilateral treatment, ipsilateral neck RT was associated with a significantly greater risk of CNF (log OR, 1.29 [95% CI, 0.09-2.48]; *P* = .04) (Figure 4). While the log OR was consistent with a 3.6-fold higher odds of CNF with ipsilateral neck RT compared with bilateral neck RT, the absolute difference in risk between the 2 approaches was small (2%). Subgroup analysis by T and N category could not be performed due to lack of information.

### Clinical and Treatment Factors Associated With CNF

To better understand factors associated with CNF, meta-regression analyses were performed. Separate univariate meta-regression models for each factor of interest were examined and the regression coefficient (β) corresponding to each factor was determined. Ultimately, none of the clinical and treatment factors examined were associated with CNF. The 2 treatment factors with the closest trend toward significance were use of IMRT (β = −0.0001 [95% CI, −0.0007 to 0.0001]; *P* = .17) (eFigure 11A in Supplement 1) and neck dissection (β = −0.0003 [95% CI, −0.0007 to 0.0001]; *P* = .11) (eFigure 11B in Supplement 1), both of which appeared to correlate with lower rates of CNF. Although not statistically significant, use of chemotherapy appeared to correlate with higher rates of CNF (β = 0.0001 [95% CI, −0.0002 to 0.0004]; *P* = .56) (eFigure 11C in Supplement 1). History of smoking, positive HPV status, median follow-up time, male sex, and median age were also not associated with CNF risk (eFigure 11D-H in Supplement 1).

### Toxic Effects Associated With Ipsilateral Neck RT

**Figure 5.**
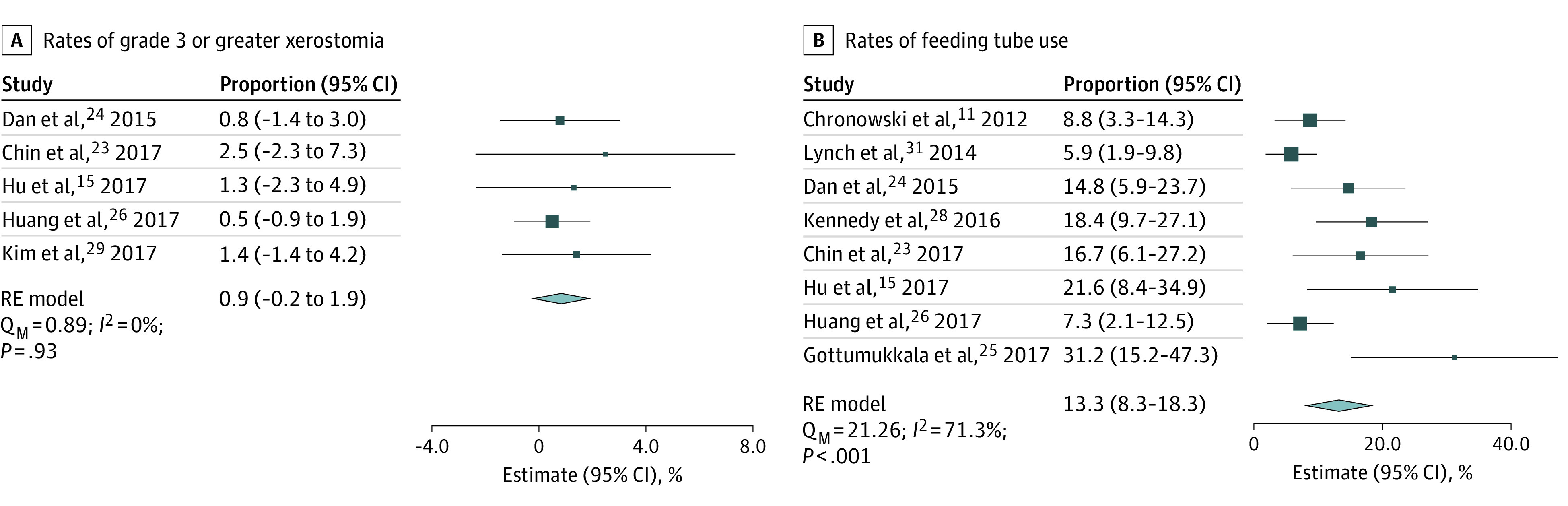
Pooled Estimates of Toxic Effects Following Ipsilateral Neck Radiotherapy Toxic effects included xerostomia of grade 3 or greater and use of feeding tube. RE indicates random-effects .

Finally, toxic effects of ipsilateral neck RT were assessed. Xerostomia of grade 3 or greater and feeding tube use were most consistently reported across included studies. The pooled rate of grade 3 or greater xerostomia after ipsilateral neck RT (n = 304) was 0.9% (95% CI, –0.2% to 1.9%; *I^2^* = 0%) (Figure 5A), while the rate of feeding tube use (n = 588) was 13.3% (95% CI, 8.3%-18.3%; *I^2^* = 71.3%) (Figure 5B). Given the high heterogeneity in pooled rate of feeding tube use, outlier studies were identified using the Cook distance. After removal of outlier studies, the estimated rate of feeding tube use was 11.6% (95% CI, 7.3%-15.9%) and heterogeneity remained high (*I^2^* = 61.5%) (eFigure 12 in Supplement 1).

## Discussion

Appropriate target selection for RT remains an area of active investigation in head and neck oncology.^36,37^ While treatment of tonsil cancers with ipsilateral neck RT was first reported over 40 years ago, omission of the contralateral uninvolved neck from the radiation field is subject to scrutiny.^33^ Both the American College of Radiology^17,18^ and American Radium Society^16^ guidelines recommend ipsilateral RT in the setting of well-lateralized primary tumors with 0 or 1 ipsilateral lymph node *(AJCC*-7 N0-N2a) involved. However, when 2 or more ipsilateral lymph nodes are involved (*AJCC*-7 N2b), bilateral neck RT is recommended: both societies cite limited evidence for ipsilateral neck RT in this situation and that it may be associated with an increased risk of CNF.

Concern for CNF following ipsilateral neck RT is reflected in clinical practice. A recent study of head and neck oncologists^38^ found that bilateral neck treatment was significantly more likely to be recommended by radiation oncologists than otolaryngologists. Similarly, the use of ipsilateral neck RT in recently published clinical trials for oropharyngeal cancer was limited.^39,40^ For example, in NRG-HN002,^39^ ipsilateral neck RT was a treatment option for patients with T1-T3N0-N2a disease or those with T1-T3N2b disease (lymph nodes only in level II) and no extranodal extension. Although 99 of the 306 enrolled patients (32%) were stratified to this treatment up front, only 37 (12%) ultimately received ipsilateral neck RT.^39^

Herein, we demonstrated that the rate of CNF following ipsilateral neck RT for tonsil cancer is low, at about 2% among all included patients. This low rate of CNF is in line with the 2 published prospective trials of ipsilateral RT in tonsil cancer: Rusthoven et al^14^ reported a CNF rate of 0 and Hu et al^15^ reported a rate of 2.7%. The CNF rate remained low even after sensitivity analysis based on study quality (eFigure 2 in Supplement 1).

Although the overall rate of CNF is low, it appears to increase with increasing T category: T3 to T4 tumors were associated with a significantly higher rate of CNF than T1 to T2 tumors. Given that T3 tumors are greater than 4 cm and T4 tumors invade adjacent structures,^41^ these tumors are more likely to have microscopic extension across midline and are therefore at increased risk for CNF following omission of the contralateral neck from the RT field.^41^ While most included studies specified that ipsilateral neck RT was used in the setting of a well-lateralized primary tumor, this definition varied by study. When comparing studies that allowed some extension toward midline against those that allowed no extension toward midline, the rate of CNF did not differ significantly.

While the risk of CNF appeared to increase by T category, it was not associated with increasing N category. Patients with N0 to N2a and N2b to N3 neck involvement had similar rates of CNF. This argues against the previously held assumption that increasing N category was associated with an increased risk of CNF due to draining lymphatics to the contralateral neck.^17,42^ To our knowledge, this study has the largest number of patients with N2b disease (n = 339) available for analysis. In this subset of patients, the CNF rate was low (3.1%). It was not associated with changes in staging system (from *AJCC*-1 to *AJCC*-7), as the definition of N2b disease remained constant over time (eTable 8 in Supplement 1). Our analysis, which included a large number of patients with N2b nodal involvement, demonstrated a low rate of CNF in this specific patient population. It should be acknowledged that the N2b classification (multiple nodes involved within the ipsilateral neck, all ≤6 cm in the largest dimension) is heterogeneous and encompasses a spectrum of disease extent from relatively limited to relatively advanced ipsilateral neck involvement. This finding likely does not indicate a broadly low risk for all patients with *AJCC*-7 N2b disease, but rather that there exists a subset of patients within this classification who have a low risk of CNF and should be considered for ipsilateral neck RT. Our analysis was unable to further define this low-risk group, but further study is warranted to this end.

Beyond tumor and nodal staging, treatment characteristics may influence outcomes following ipsilateral neck RT. While none of the factors examined were associated with CNF, use of IMRT and neck dissection demonstrated the closest trend toward significance, and both appeared to be associated with lower rates of CNF. One possible reason for this finding is that IMRT is a more modern RT modality that enables better target delineation. As a result, the likelihood of a geographic miss, which could result in disease recurrence, would be decreased. It is also likely that advances in diagnostic imaging occurring during the adoption of IMRT allowed for this more modern patient population to undergo accurate clinically staging, thus ruling out occult contralateral disease that could manifest as CNF after ipsilateral neck RT. While the laterality of neck dissection was not specified, contralateral neck dissection, in theory, could eliminate occult disease within the contralateral neck and thus contribute to lower rates of CNF. Finally, while chemotherapy use was not associated with CNF, chemotherapy use appeared to be correlated with higher rates of CNF. This potential association could represent a selection bias, given that concurrent chemotherapy is typically used in patients with more advanced tumors or nodal disease.

Despite concern for CNF with ipsilateral neck RT, randomized prospective comparisons with bilateral neck RT are lacking. Results of retrospective analyses were mixed, with some studies showing a benefit of bilateral neck RT over ipsilateral neck RT, while others showed similar outcomes.^23,29,30,32,33,35^ To our knowledge, the analysis reported herein is the largest comparison between the 2 treatments. While we demonstrated a relative benefit of bilateral neck RT compared with ipsilateral neck RT (3.6-fold higher odds with ipsilateral RT), the absolute benefit was small (2%) and the significance level was marginal (*P* = .04). Moreover, due to limitations in available data, further subgroup analyses by T and N category were not possible, nor was it possible to propensity match the 2 groups.

Compared with bilateral neck RT, ipsilateral neck RT is associated with a lower burden of acute and late radiation-induced toxic effects. A prospective study comparing ipsilateral and bilateral neck RT in patients with oropharyngeal cancer^43^ found that ipsilateral treatment was associated with improved clearing of the vallecula and pyriform sinus and decreased aspiration. In our study, the 2 most commonly reported adverse events were grade 3 or greater xerostomia and use of a feeding tube. While timing of these events (acute vs late) was not specified, the pooled rate of grade 3 or greater xerostomia was 0.9%, while the rate of feeding tube use was 13.3%. Although the heterogeneity of the rate of feeding tube use was high, even after removal of outlier studies, we believe this reflects underlying variability in clinical practice. For example, policies for feeding tube placement are institution dependent, and supportive services (eg, dedicated nutritionists) may not be available. These findings are in line with those of NRG-HN002, where acute and late grades 2 to 3 xerostomia was reported in 51% and 26% of patients treated with chemoradiotherapy, respectively.^39^ Feeding tube rates 1 month after completion of RT were as high as 22%, and the 95% CI around this metric was relatively wide (approximately 10% in either direction).^39^ Given that most patients in this trial were treated with bilateral neck RT, our findings indicated that ipsilateral neck RT may reduce the risk of radiation-induced adverse events.

### Strengths and Limitations

Overall, our study had several strengths. First, to our knowledge, this is the largest analysis of outcomes following ipsilateral neck RT for tonsil cancer. The patient population is heterogeneous and spans both the eras in which oropharyngeal SCC was predominantly negative for HPV and, subsequently, predominantly associated with HPV. The included studies also span multiple decades where different clinical practices (eg, use of diagnostic imaging, staging edition, etc) were used. We addressed factors that could contribute to heterogeneity by performing sensitivity analyses and meta-regression. We found no association between CNF and multiple clinical and treatment-related factors, including use of diagnostic imaging, clinical staging edition, degree of midline extension, HPV status, chemotherapy use, use of neck dissection, smoking history, or use of IMRT. Additionally, this analysis was designed prospectively, used appropriate statistics, and can be used to power future randomized studies.

This study does, however, have some limitations. Most studies included in our analysis were retrospective. Because we conducted a study-level meta-analysis and not a patient-level analysis, detailed comparisons between subgroups were not possible. Care should be taken when interpreting these findings in the context of the more recent *AJCC*-8 staging system, in which HPV-associated oropharyngeal SCC has a different nodal staging than non–HPV-associated disease, and the N2 designation now refers to the presence of bilateral or contralateral lymph node involvement (previously N2c in *AJCC*-7). Moreover, meta-regression analyses are hypothesis-generating and require additional validation. Finally, certain risk factors of clinical and prognostic significance (eg, extranodal extension) were not addressed due to limited available data. We were also unable to compare toxic effects or patient-reported outcomes by whether patients received ipsilateral or bilateral neck RT. This highlights the need for complete and consistent reporting of toxic effects and patient-reported outcome measures in RT studies of patients with head and neck cancer.

Ultimately, the findings of our systematic review and meta-analysis further inform the risk-benefit discussion between physicians and patients with tonsil cancer. By limiting the radiation target, ipsilateral neck RT has the potential to improve treatment toxic effects and patient-reported outcomes. While concern for failure is valid, our study demonstrates that the risk of CNF is lower than what is commonly assumed. In other disease sites (eg, breast cancer), omission of RT is an area of active investigation, particularly when the risk of recurrence is low (<10%-15%). Historically, salvage RT to the contralateral neck was technically difficult and morbid. However, newer RT technologies (eg, IMRT, protons) allow for more conformal dose distribution and reduce overlap with prior RT fields. While a prospective, randomized trial is needed to best assess differences between bilateral and ipsilateral RT, our findings allow for more comprehensive decision-making for patients with tonsil cancer.

## Conclusions 

For patients with small, lateralized tonsil cancers, ipsilateral neck RT is associated with a low rate of CNF in this systematic review and meta-analysis. The risk of CNF appears to increase with increasing T category but not with increasing N category. Compared with ipsilateral treatment, bilateral neck RT is associated with a small, but significant, improvement in CNF rate. This study provides the strongest level of evidence to date to support the use of ipsilateral neck RT in patients with well-lateralized tonsil cancers, including those with 2 or more ipsilateral lymph nodes.

## Notes

Supplement 1. **eMethods.** Search Strategy, Statistical Methods, and Systematic Review Results **eFigure 1.** Funnel Plot for Publication Bias for Contralateral Neck Failure **eFigure 2.** Contralateral Neck Failure Rate Based on Study Quality **eFigure 3.** Contralateral Neck Failure Rates Grouped by Staging Edition **eFigure 4.** Contralateral Neck Failure Rates Grouped by Diagnostic Imaging **eFigure 5.** Contralateral Neck Failure Rates Grouped by T Stage **eFigure 6.** Comparing Contralateral Neck Failure by T Stage **eFigure 7.** Contralateral Neck Failure Rates Grouped by Primary Tumor Extension **eFigure 8.** Contralateral Neck Failure Rates Grouped by N Stage **eFigure 9.** Comparing Contralateral Neck Failure by N Stage **eFigure 10.** Contralateral Neck Failure Rates Grouped by RT Type **eFigure 11.** Association Between Treatment and Risk Factors With Contralateral Neck Failure **eFigure 12.** Heterogeneity After Excluding Outlier Feeding Tube Studies **eTable 1.** Treatment Characteristics of Studies Included for Meta-analysis **eTable 2.** Baseline Characteristics of Studies Included for Meta-analysis **eTable 3.** T and N Stages of Studies Included for Meta-analysis **eTable 4.** Contralateral Neck Failure (CNF) Outcomes of Included Studies by RT Type **eTable 5.** Contralateral Neck Failure (CNF) Following Ipsilateral RT by T and N Stages **eTable 6.** Toxicity Outcomes—Grade 3 or Greater Xerostomia and G Tube Use **eTable 7.** Quality of Included Studies per MINORS Criteria **eTable 8.** Definition of N2b Disease by Staging Edition
eReferences

Supplement 2.
Data Sharing Statement
