# Supplementary material for: Association of Unilateral Radiotherapy With Contralateral Lymph Node Failure Among Patients With Squamous Cell Carcinoma of the Tonsil: A Systematic Review and Meta-analysis
Source: JAMA Netw Open. 2023 Feb 8;6(2):e2255209. doi: 10.1001/jamanetworkopen.2022.55209 (PMC9909500; doi:10.1001/jamanetworkopen.2022.55209)
Supplement: Supplement 1. — eMethods. Search Strategy, Statistical Methods, and Systematic Review Results eFigure 1. Funnel Plot for Publication Bias for Contralateral Neck Failure eFigure 2. Contralateral Neck Failure Rate Based on Study Quality eFigure 3. Contralateral Neck Failure Rates Grouped by Staging Edition eFigure 4. Contralateral Neck Failure Rates Grouped by Diagnostic Imaging eFigure 5. Contralateral Neck Failure Rates Grouped by T Stage eFigure 6. Comparing Contralateral Neck Failure by T Stage eFigure 7. Contralateral Neck Failure Rates Grouped by Primary Tumor Extension eFigure 8. Contralateral Neck Failure Rates Grouped by N Stage eFigure 9. Comparing Contralateral Neck Failure by N Stage eFigure 10. Contralateral Neck Failure Rates Grouped by RT Type eFigure 11. Association Between Treatment and Risk Factors With Contralateral Neck Failure eFigure 12. Heterogeneity After Excluding Outlier Feeding Tube Studies eTable 1. Treatment Characteristics of Studies Included for Meta-analysis eTable 2. Baseline Characteristics of Studies Included for Meta-analysis eTable 3. T and N Stages of Studies Included for Meta-analysis eTable 4. Contralateral Neck Failure (CNF) Outcomes of Included Studies by RT Type eTable 5. Contralateral Neck Failure (CNF) Following Ipsilateral RT by T and N Stages eTable 6. Toxicity Outcomes—Grade 3 or Greater Xerostomia and G Tube Use eTable 7. Quality of Included Studies per MINORS Criteria eTable 8. Definition of N2b Disease by Staging Edition eReferences [file jamanetwopen-e2255209-s001.pdf]

Supplementary Online Content

Razavian NB, D'Agostino RB Jr, Steber CR, Helis CA, Hughes RT. Association of unilateral radiotherapy with contralateral lymph node failure among patients with squamous cell carcinoma of the tonsil: a systematic review and meta-analysis. *JAMA Netw Open*. 2023;6(2):e2255209. doi:10.1001/jamanetworkopen.2022.55209

eMethods. Search Strategy, Statistical Methods, and Systematic Review Results
eFigure 1. Funnel Plot for Publication Bias for Contralateral Neck Failure
eFigure 2. Contralateral Neck Failure Rate Based on Study Quality
eFigure 3. Contralateral Neck Failure Rates Grouped by Staging Edition
eFigure 4. Contralateral Neck Failure Rates Grouped by Diagnostic Imaging
eFigure 5. Contralateral Neck Failure Rates Grouped by T Stage
eFigure 6. Comparing Contralateral Neck Failure by T Stage
eFigure 7. Contralateral Neck Failure Rates Grouped by Primary Tumor Extension
eFigure 8. Contralateral Neck Failure Rates Grouped by N Stage
eFigure 9. Comparing Contralateral Neck Failure by N Stage
eFigure 10. Contralateral Neck Failure Rates Grouped by RT Type
eFigure 11. Association Between Treatment and Risk Factors With Contralateral Neck Failure
eFigure 12. Heterogeneity After Excluding Outlier Feeding Tube Studies
eTable 1. Treatment Characteristics of Studies Included for Meta-analysis
eTable 2. Baseline Characteristics of Studies Included for Meta-analysis
eTable 3. T and N Stages of Studies Included for Meta-analysis
eTable 4. Contralateral Neck Failure (CNF) Outcomes of Included Studies by RT Type
eTable 5. Contralateral Neck Failure (CNF) Following Ipsilateral RT by T and N Stages
eTable 6. Toxicity Outcomes—Grade 3 or Greater Xerostomia and G Tube Use
eTable 7. Quality of Included Studies per MINORS Criteria
eTable 8. Definition of N2b Disease by Staging Edition
eReferences

This supplementary material has been provided by the authors to give readers additional information about their work.

© 2023 Razavian NB et al. *JAMA Network Open*.

eMethods. Search Strategy, Statistical Methods, and Systematic Review Results

Search Strategy
Three electronic databases (PubMed, Embase, Web of Science, and Cochrane Library) were queried for published articles from 01/01/1980 to 12/31/2021. Searches of PubMed, Embase, and Web of Science were performed on 01/15/22, while search of Cochrane Library was performed on 11/29/22.

The following search terms were used to query each database:
"Tonsil" OR "tonsillar" OR "tonsils" OR "oropharynx" OR "oropharyngeal") AND ("Neoplasm" OR "neoplasms" OR "carcinoma" OR "carcinomas" OR "Cancer" OR "Cancers") AND ("radiotherapy" OR "Radiotherapies" OR "Radiation Therapy" OR "Targeted Radiation Therapies" OR "radiation" OR "RT" OR "irradiation" OR "UNI" OR "IMRT") AND ("unilateral" OR "lateralized" OR "laterality" OR "ipsilateral" OR "lateral"

Articles were included for analysis if they were peer reviewed and contained 20 or more patients treated with ipsilateral neck irradiation for tonsil cancer. We used ≥20 patients as a size cutoff in order to minimize bias, heterogeneity, and underestimation of results from smaller studies. Articles were reviewed by 2 or more authors and discrepancies were resolved by a consensus committee consisting of 3 authors. References within each included article were also searched (i.e. snowballing). Given that the topic of this systematic review was a clinical question, grey literature search for non-peer reviewed studies, non-governmental or international governmental documents, etc, were not performed. The Cochrane library database includes searches of clinicaltrials.gov and other international clinical trial databases.

Full inclusion and exclusion criteria per PICOTS format:

<u>Population</u>: Included peer reviewed studies treated 20 or more patients with squamous cell carcinoma of the tonsil cancer. Patients of all genders, race socioeconomic status, and comorbidities were eligible for inclusion. Studies treating patients with other oropharyngeal tumors (e.g. base of tongue, soft palate) were excluded.

<u>Intervention</u>: Included studies treated patients with unilateral neck RT. Studies with omission of only one lymph node level (e.g. retropharyngeal nodes), patients with bilateral neck involvement, lack of radiation details, or only reporting results after bilateral neck RT were excluded.

<u>Comparator</u>: This study was designed to describe rates of contralateral neck failure (CNF) after unilateral neck RT and thus no control group was specified for the primary end point. However, for studies that reported outcomes of bilateral neck RT, this was considered the comparator treatment for secondary analysis (see below).

<u>Outcomes</u>: Primary outcome was the pooled rate of CNF following ipsilateral neck RT. CNF was defined as a nodal failure within the contralateral, unirradiated, neck as determined by clinical, pathologic, or radiographic assessments. Secondary outcomes included pooled rates of CNF by T-stage, N-stage, use of bilateral RT, and toxicity. Pooled rates were estimated using random-effects model. Pre-planned subgroup analyses included comparisons of CNF by T-stage and N-stage. Additional analyses examined the association of CNF with clinical and treatment factors such as use of diagnostic imaging, degree of midline tumor extension, staging edition used, RT treatment modality, HPV status, smoking status, use of surgery, and use of chemotherapy.

<u>Timing</u>: Included studies treated patients with radiation in either a definitive or adjuvant (post-operative) manner. Patient follow-up was performed per institutional standard of care.

<u>Setting</u>: Included studies consisted of patients treated by radiation oncologists, which is generally delivered in the outpatient setting.

Statistical Methods
After identifying studies included for statistical analysis, data extraction was performed by 2 authors. In cases of discrepancy, consensus was established among a committee of 3 authors. Multiple variables were extracted including numbers of patients receiving ipsilateral neck RT, bilateral neck RT, N-stage, T-stage, staging edition, diagnostic imaging use, degree of midline extension, chemotherapy use, smoking history, use of neck dissection, use

© 2023 Razavian NB et al. *JAMA Network Open*.

of IMRT, follow-up time, and HPV status. Only data available within each manuscript was used for statistical analysis. Given the consensus agreement for study selection and data extraction, kappa statistics were not performed.

Study quality was assessed using MINORS criteria. Using this scale, retrospective studies can have a maximum score of 12, while prospective studies can have a maximum score of 14. We considered lower quality studies to be those with scores below the first quartile.

Publication bias was assessed with funnel plots of sample size. Publication bias was tested via Egger's regression test using the *regtest* function with the *metafor* package in R.

Pooled estimates of each outcome of interest (e.g. CNF) were calculated using a random-effects model in which the proportion of each outcome was weighted inversely by study size. Proportions were used because we were interested in estimating the percentage of patients that would develop a given outcome of interest. Event counts were not used as person-time data were not available. More specifically, the number of patients experiencing an event (e.g. CNF) and the total number of patients at risk for an event (e.g. patients receiving ipsilateral neck RT) were used as inputs for the *escalc* functions within the *metafor* package. Using the programs default settings, the raw proportion of each event was calculated. Transformations (e.g. logit, log, arcsine square root) were not used. In the case of proportions equal to zero, a value of 0.5 was used in the measurement of effect size as per the *escalc* default settings. These data were subsequently input into the *rma* function in order to calculate a pooled estimate of the outcome of interest based on a random-effects model.

A random-effects model was chosen for several reasons. Given that the included studies span multiple decades and institutions, heterogeneity was expected. Compared to a fixed-effects model, the random-effects model provides wider confidence intervals and thus a more conservative estimate of each outcome. Additionally, the assumptions underlying a random-effects model were felt to be more appropriate in this clinical setting – i.e. the observed differences among studies were due to both underlying variation (in treatment setting, institution, etc) and random chance.

Heterogeneity of each pooled analysis was estimated using the Cochrane's Q test and the Higgins I^2. Heterogeneity was considered to be "high" when I^2 was >50% or the P-value from Cochrane's Q test was <0.05. In cases of high heterogeneity, Cook's distance was calculated to identify outlier studies using *influence* function in the *metafor* package in R. A sensitivity analysis was then performed by removing the outlier studies and re-calculating the pooled estimate and heterogeneity.

All comparisons between groups were performed using the *escalc* and *rma* functions with the *metafor* package in R. Two-group comparisons of patient level variables within each study were performed using the log-odds ratio. For these analyses, studies were only included if they reported the outcome of interest (e.g. CNF) for each variable being compared (e.g. CNF in patients with T1-2 vs CNF in patients T3-4). The log-odds ratio was used as these were dichotomous variables and person-time data were not available. Calculations were performed as follows: for each study the number of patients in each group that developed the outcome of interest (e.g. CNF in patients with T1-2 disease) and the number of patients at risk in each group at risk for the outcome of interest (e.g. patients with T1-2 disease receiving ipsilateral neck RT) were input into the *escalc* function and the odds ratio between the two groups and effects size were calculated. Per default settings, the odds ratio was log transformed (making the outcome symmetric around zero) in order to make sample distributions better approximate normality. These data were then used as inputs for the *rma* function, which estimated a pooled log-odds ratio using a random-effects model. Pooled estimates greater than 0 indicate that the odds of the outcome of interest (e.g. CNF) are greater in the comparison group (e.g. T3-4 disease) than the control group (e.g. T1-2 disease), while estimates less than 0 indicate that the odds of the outcome of interest are lower in the comparison group than in the control group.

For comparisons of CNF between study level variables (e.g. studies that used diagnostic imaging vs studies that did not use diagnostic imaging), the omnibus test of moderators (Q_M) was performed through the *escalc* and *rma* function within *metafor* using default settings. Here, a mixed-effects meta-regression model was constructed that includes each defined subgroup as a predictor of CNF. The null hypothesis of the omnibus test was that all subgroups are unrelated to CNF. The Q_M test follows asymptotically a chi-square distribution with *m* degrees of freedom (with *m* denoting the number of subgroups examined). When the P-value of the Q_M statistics is <0.05, this indicates that the variance in pooled estimate of one subgroup is significantly greater than what can be expected by chance, and thus that this subgroup is associated with CNF.

Comparisons using proportions were performed to better understand how different clinical/patient factors (e.g. IMRT use) were associated with CNF. Proportions were used in these cases because data was not available to definitively ascribe CNF by each patient/clinical factor. For these analyses, the proportion of patients with a given clinical factor (e.g. % patients receiving IMRT) and the proportion of patients with CNF (% patients developing

© 2023 Razavian NB et al. *JAMA Network Open*.

CNF) for each study was recorded. Effect size was measured using the *escalc* function in the *metafor* package. These data were then input into the *rma* function, which constructs a mixed-effects meta-regression model. For each factor examined, a regression co-efficient (β) and P-value are reported. The sign of the regression co-efficient indicates the direction of association.

Systematic Review Results

Studies were excluded from final analysis if they were case reports or series (158 articles), dosimetry studies (164 articles), conference abstracts (117 articles), surgical series (105 articles), outside the scope of this review (108 articles), focused on other neoplasms or non-neoplastic conditions (92 articles), used bilateral irradiation techniques (88 articles), were written in a foreign language (73 articles), reviews (52 articles), did not provide radiation treatment (49 articles), radiology studies (36 studies), did not provide complete radiation details (18 articles), full text not available for review (7 articles), active clinical trial (25 studies), did not specify tumor location in oropharynx (7 articles), treatment in the salvage setting (5 articles), or had overlapping patient populations (4 articles).

During full text review, snowballing did not yield additional citations. A total of 4 articles were excluded due to overlapping patient populations. This was done to reduce confounding from duplicate patients. In these situations, the larger of the studies were included for analysis. The articles excluded for this reasons include: Rusthoven et al (Int J Radiat Oncol Biol Phys, 2009), which overlaps with Dan et al (2015); Mendenhall et al (Am J Clin Oncol, 2006) and Kennedy et al (Eur Arch Otohinolaryngol, 2016), which overlap with Kennedy et al (2016); and Koo et al (Radiat Oncol J, 2013), which overlaps with Kim et al (2017).

Additionally, 4 studies that underwent full text review had <20 patients treated with ipsilateral neck RT. In all cases, the studies were excluded for reasons other than size. Specifically, 1 study (Gallitto et al, Head Neck, 2019) had incomplete treatment details and did not specify outcomes for patients treated with ipsilateral neck RT. For the other 3 studies (Longton et al, Int J Radiat Oncol Biol Phys, 2020; Vergeer et al, Int J Radiat Oncol Biol Phys, 2010; Murakami et al, BMC Cancer, 2016), while treatment outcomes following ipsilateral neck RT were provided, these studies treated multiple types of oropharyngeal tumors (tonsil, base of tongue, soft palate, etc) and did not specify whether CNFs were among patients with tonsil tumors or those with tumors in other oropharyngeal locations.

© 2023 Razavian NB et al. *JAMA Network Open*.

eFigure 1. Funnel Plot for Publication Bias for Contralateral Neck Failure

Funnel plot displays rates of CNF across all included studies. P-value is from Egger's regression test for funnel plot symmetry. P-value >0.05 indicates no statistically significant publication bias was identified.

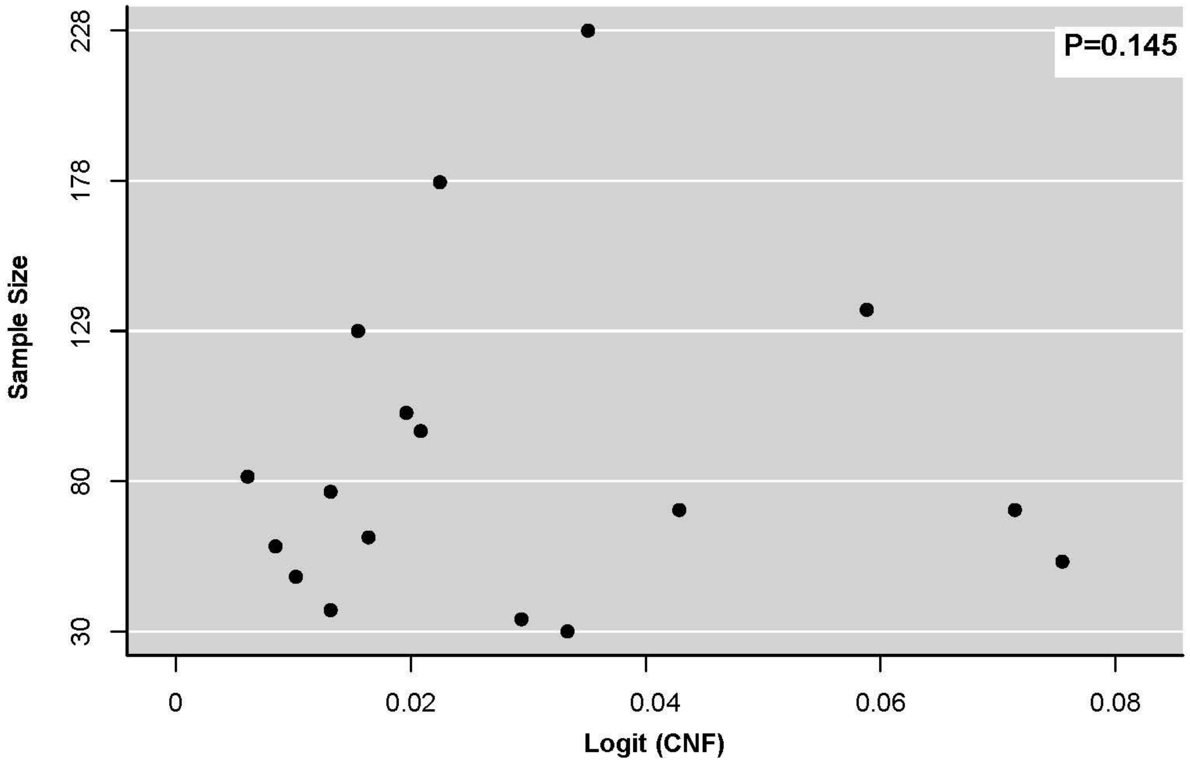

© 2023 Razavian NB et al. *JAMA Network Open*.

eFigure 2. Contralateral Neck Failure Rate Based on Study Quality

MINORS criteria was used to assess quality of included studies. Using this scale, retrospective studies can have a maximum score of 12, while prospective studies can have a maximum score of 14. Lower quality studies were considered to be those with score below the first quartile. Among all included study the median score by MINORS criteria was 10. Two studies (Murthy et al and Jackson et al) were lower quality (both with scores of 7). Pooled rates of CNF were re-estimated with the lower quality studies (n=2) removed. The resulting pooled estimate of CNF was unchanged.

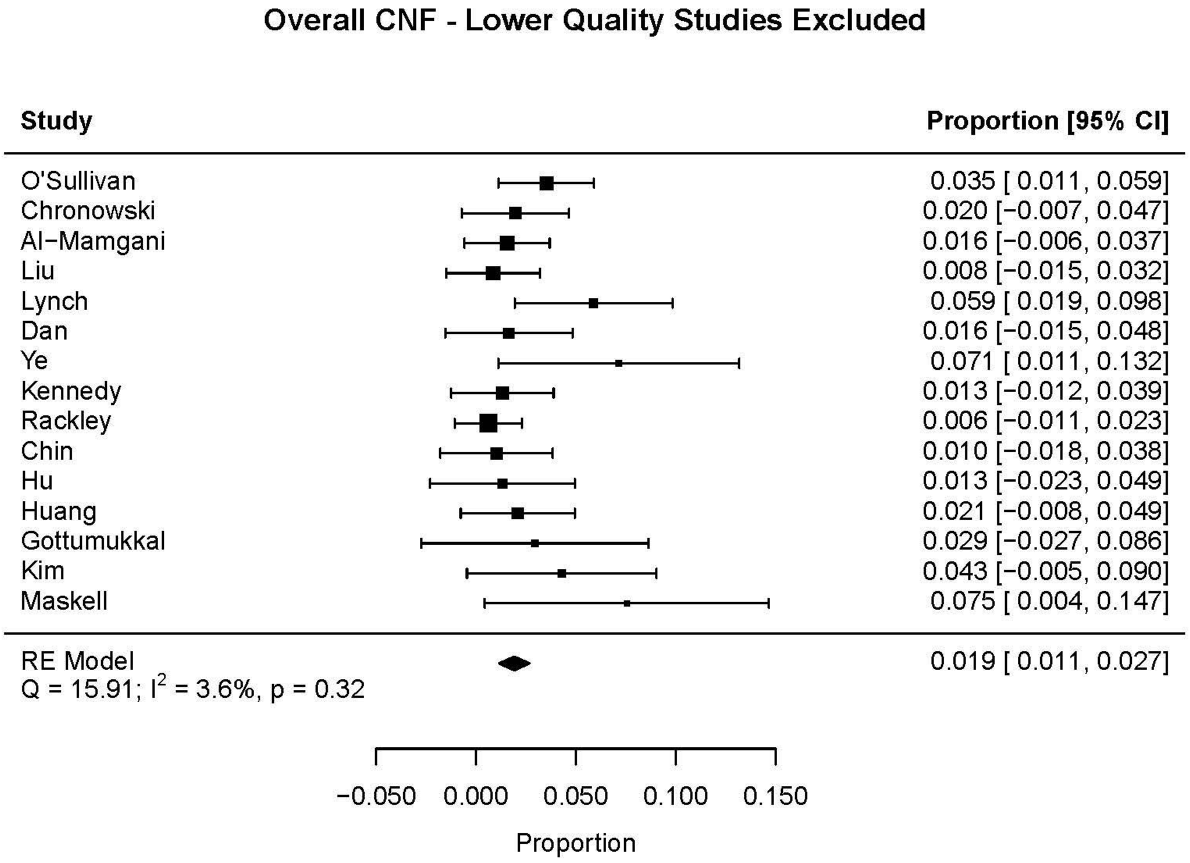

© 2023 Razavian NB et al. *JAMA Network Open*.

eFigure 3. Contralateral Neck Failure Rates Grouped by Staging Edition

Pooled contralateral neck failure outcomes are shown grouped by staging edition employed by each included publication: AJCC 7th edition, earlier (AJCC 1st-5th) staging editions, no staging edition provided. There was no significant difference in CNF by staging edition (P=0.57).

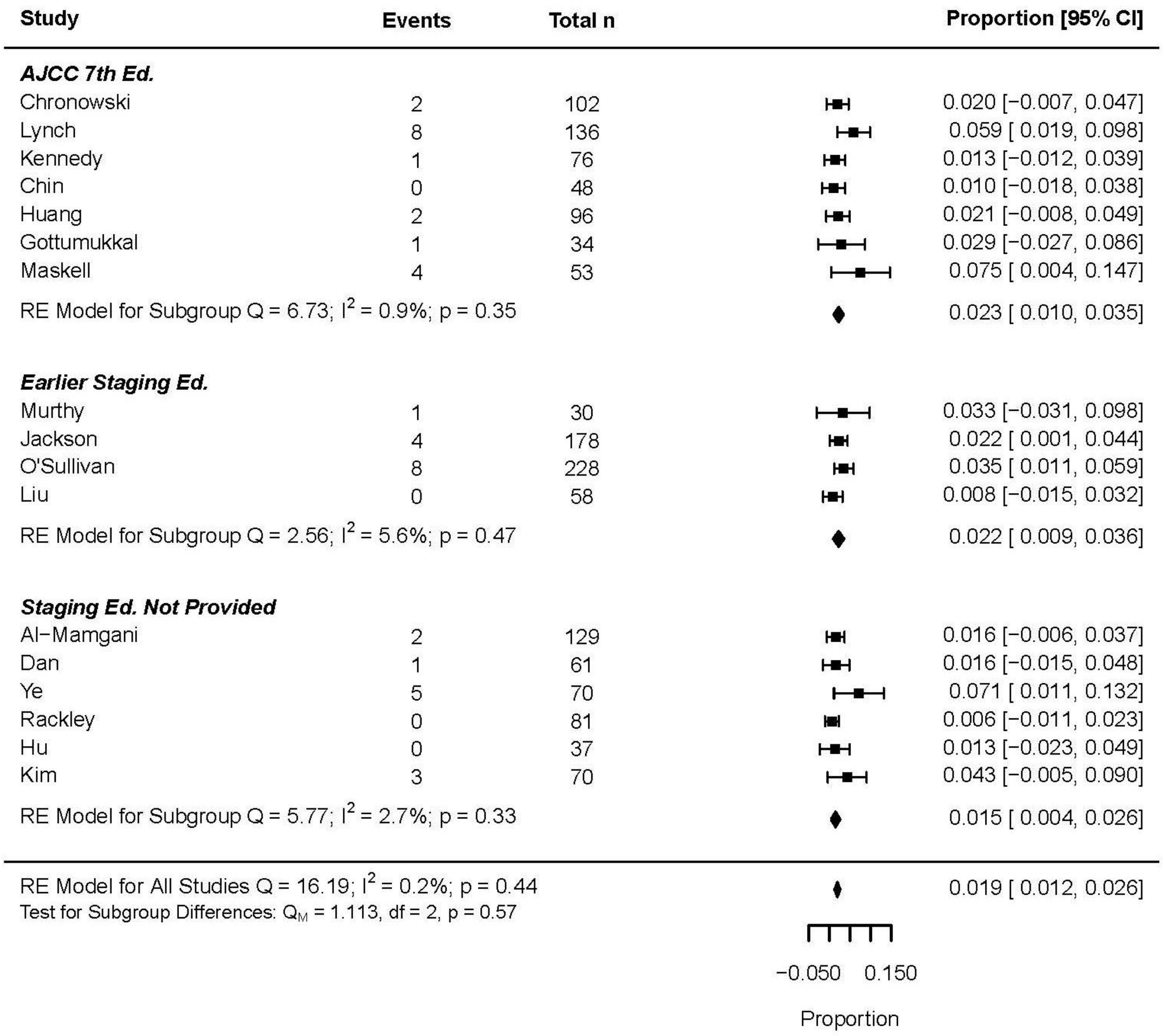

© 2023 Razavian NB et al. *JAMA Network Open*.

eFigure 4. Contralateral Neck Failure Rates Grouped by Diagnostic Imaging

Pooled contralateral neck failure outcomes are shown grouped by use of diagnostic imaging. There was no significant difference in CNF by use of diagnostic imaging (P=0.27).

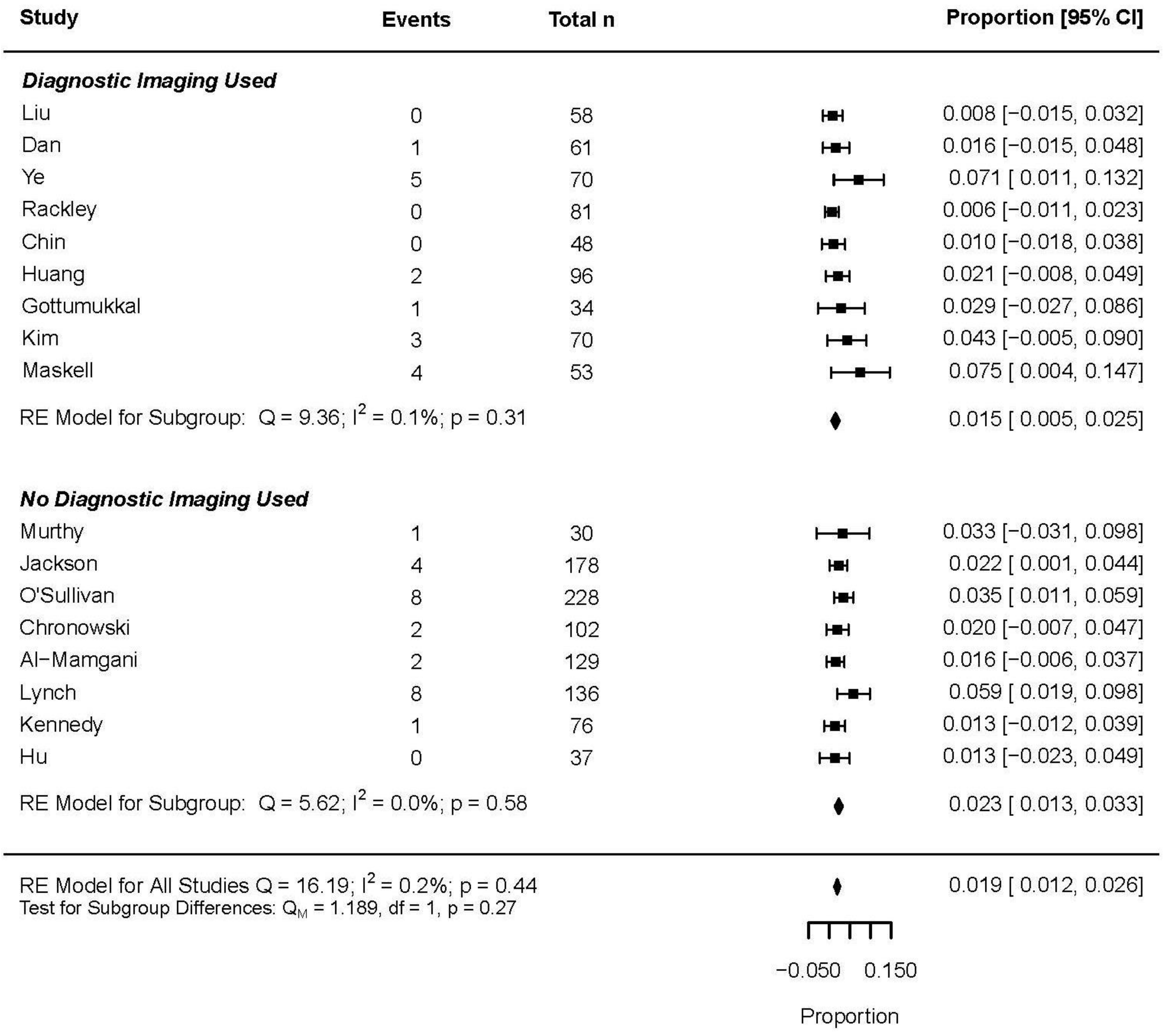

© 2023 Razavian NB et al. *JAMA Network Open*.

eFigure 5. Contralateral Neck Failure Rates Grouped by T Stage

Pooled contralateral neck failure outcomes are shown grouped by T-stage: (A) T1, (B) T2, (C) T3, (D) T4.

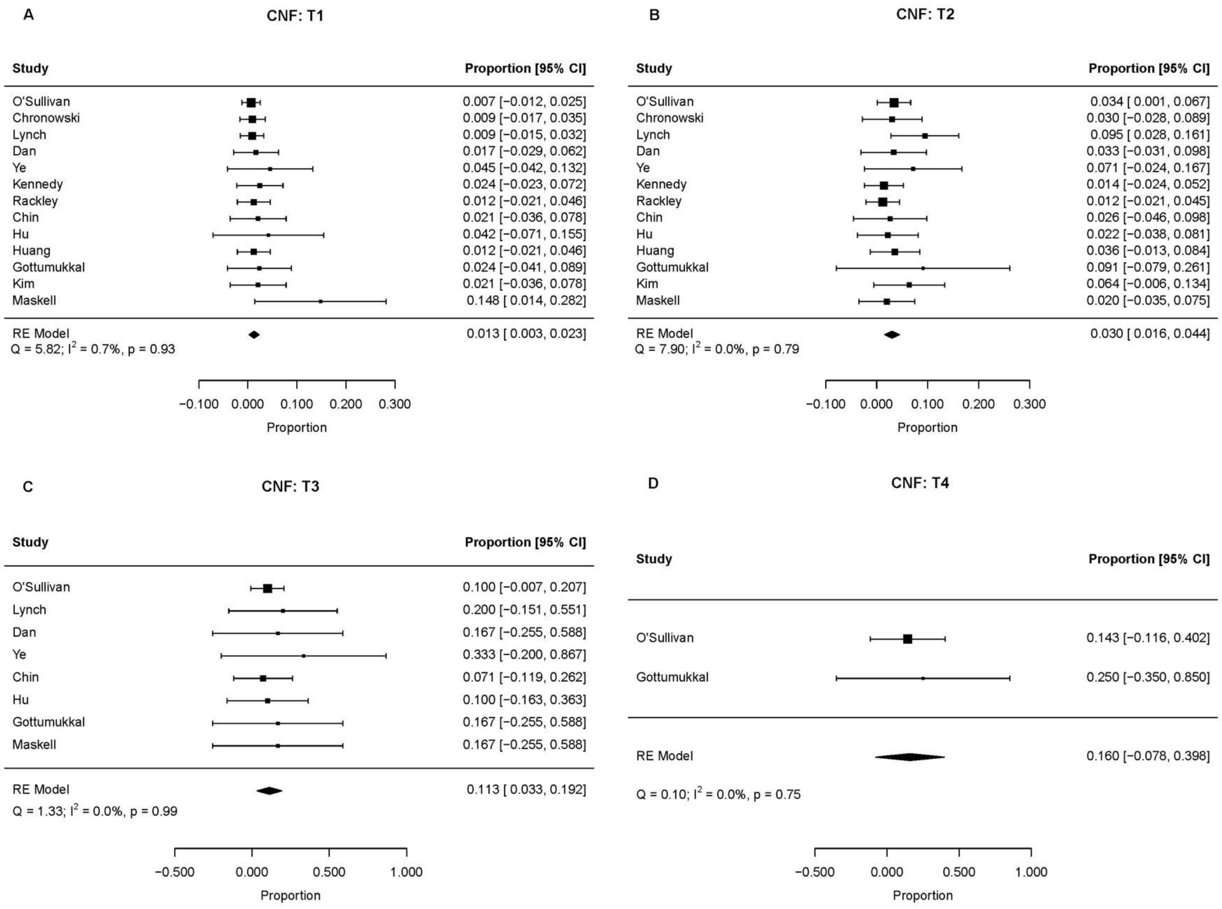

© 2023 Razavian NB et al. *JAMA Network Open*.

eFigure 6. Comparing Contralateral Neck Failure by T Stage

Odds of contralateral neck failure between T1-2 versus T3-4 disease were estimated using the log odds ratio. Ratio >1 indicates greater odds of CNF in patients with T3-4 disease. There was a significantly greater odds of developing CNF with T3-4 disease (P<0.001).

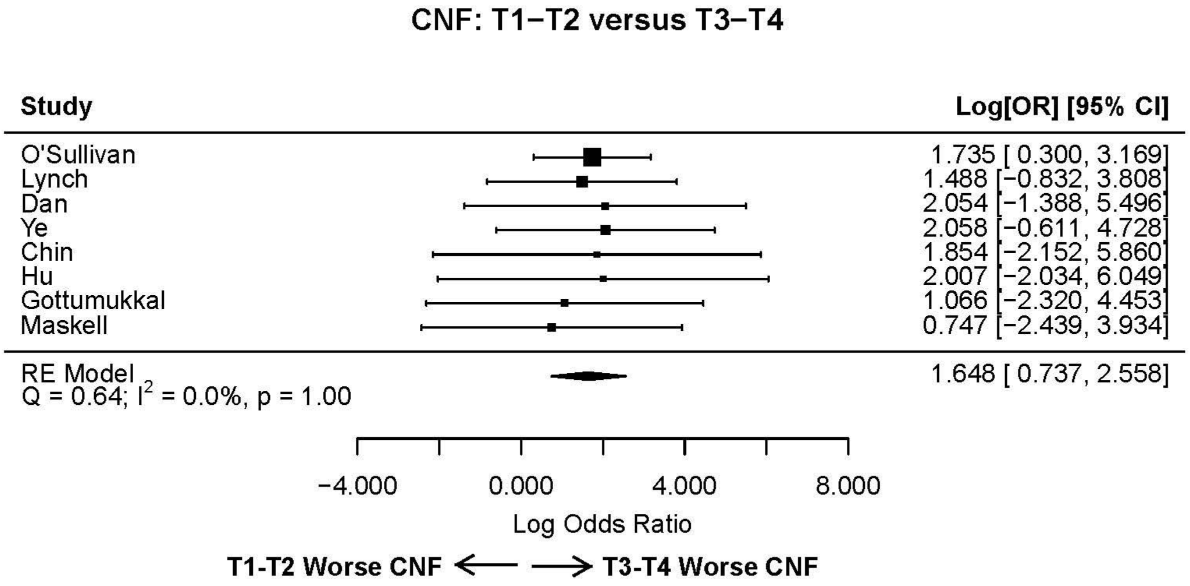

© 2023 Razavian NB et al. *JAMA Network Open*.

eFigure 7. Contralateral Neck Failure Rates Grouped by Primary Tumor Extension

Pooled contralateral neck failure outcomes are shown grouped by degree of primary tumor extension towards midline: some extension allowed, no midline extension, no information provided. There was no significant difference in CNF by degree of primary tumor extension towards midline (P=0.06).

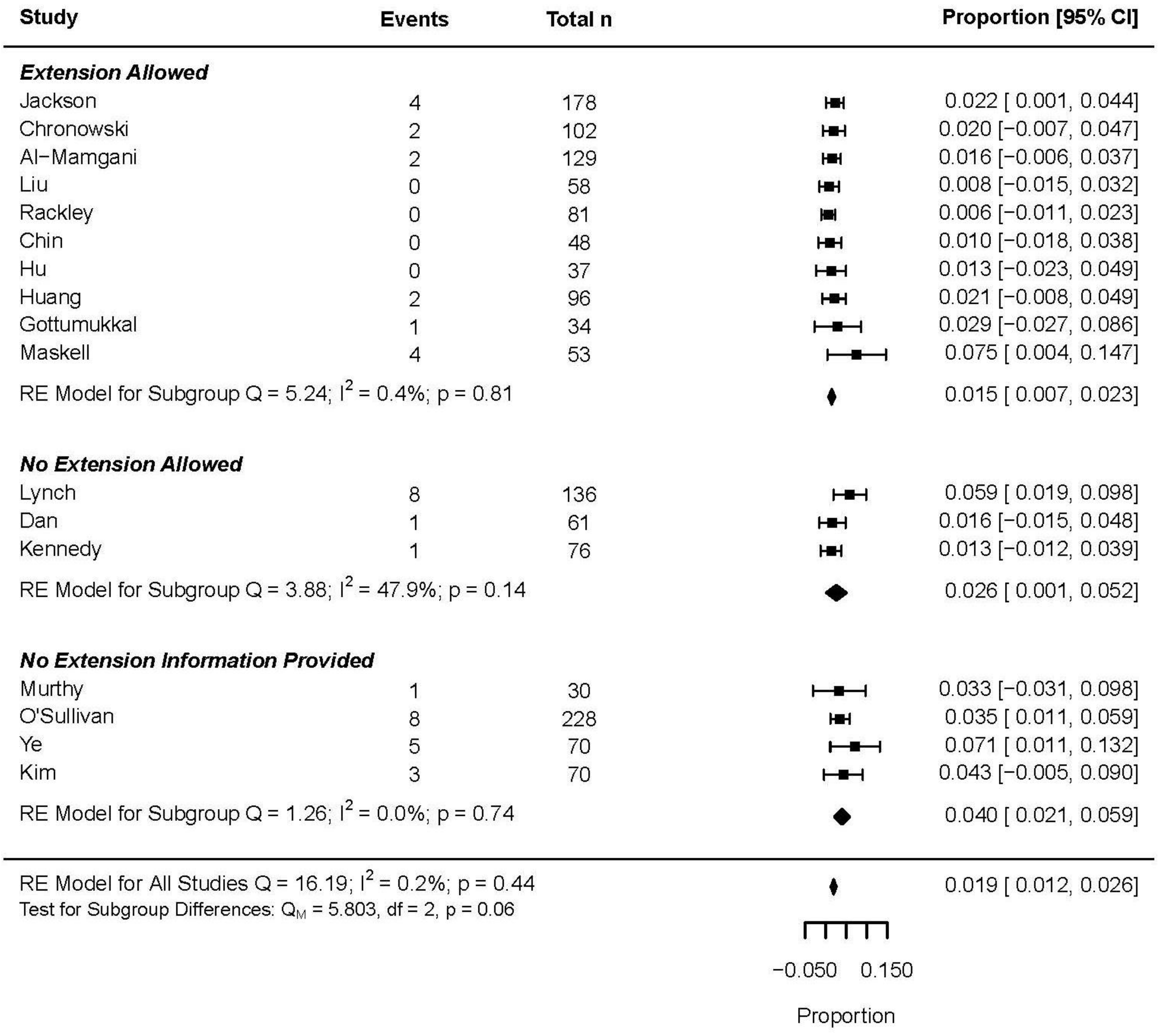

© 2023 Razavian NB et al. *JAMA Network Open*.

eFigure 8. Contralateral Neck Failure Rates Grouped by N Stage

Pooled contralateral neck failure outcomes are shown grouped by N-stage: (A) N0, (B) N1, (C) N2a, (D) N2b.

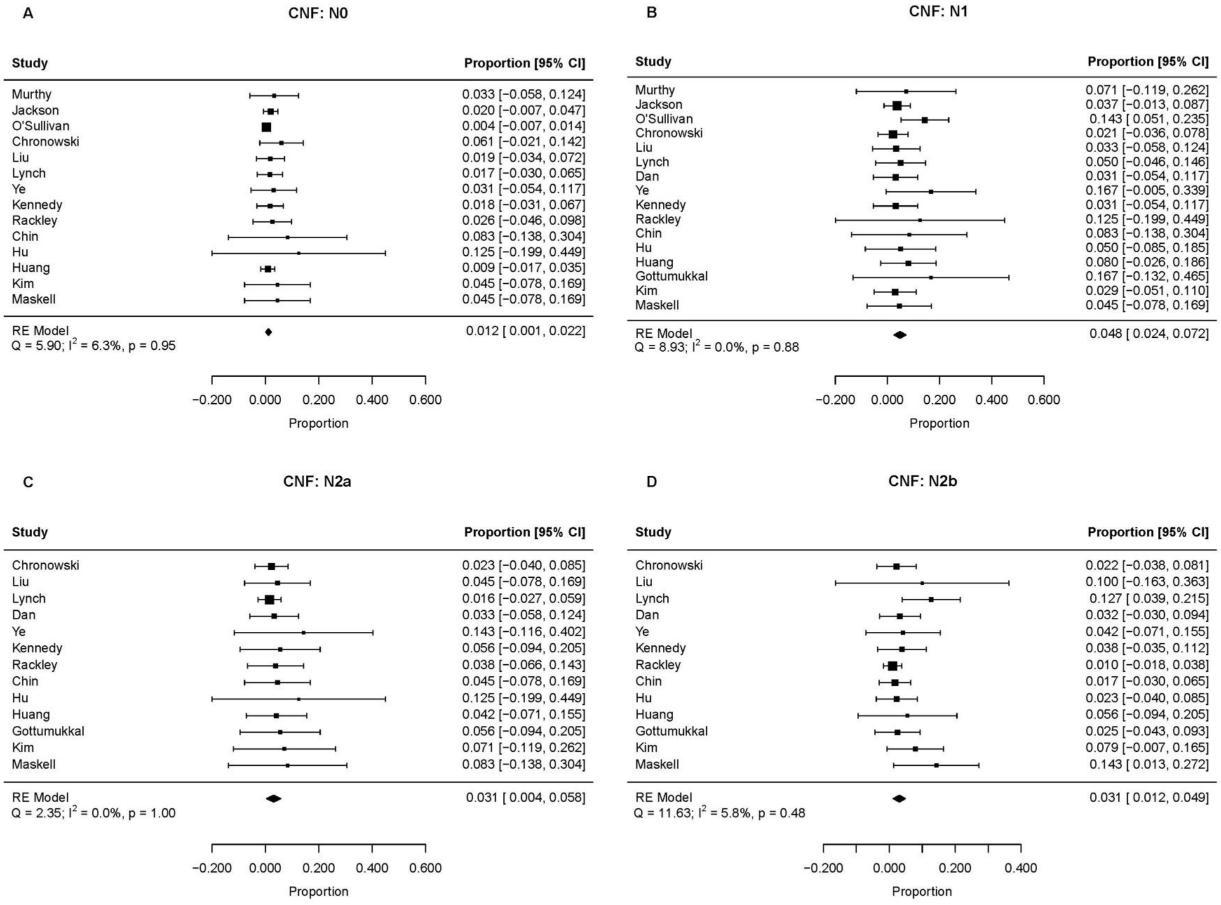

© 2023 Razavian NB et al. *JAMA Network Open*.

eFigure 9. Comparing Contralateral Neck Failure by N Stage

Odds of contralateral neck failure between N0-2a versus N2b-3 disease were estimated using the log odds ratio. Ratio >1 indicates greater odds of CNF in patients with N2b-N3 disease. There was no significant difference in odds of developing CNF in patients with N2b-N3 disease compared to patients with N0-N2a disease (P=0.072).

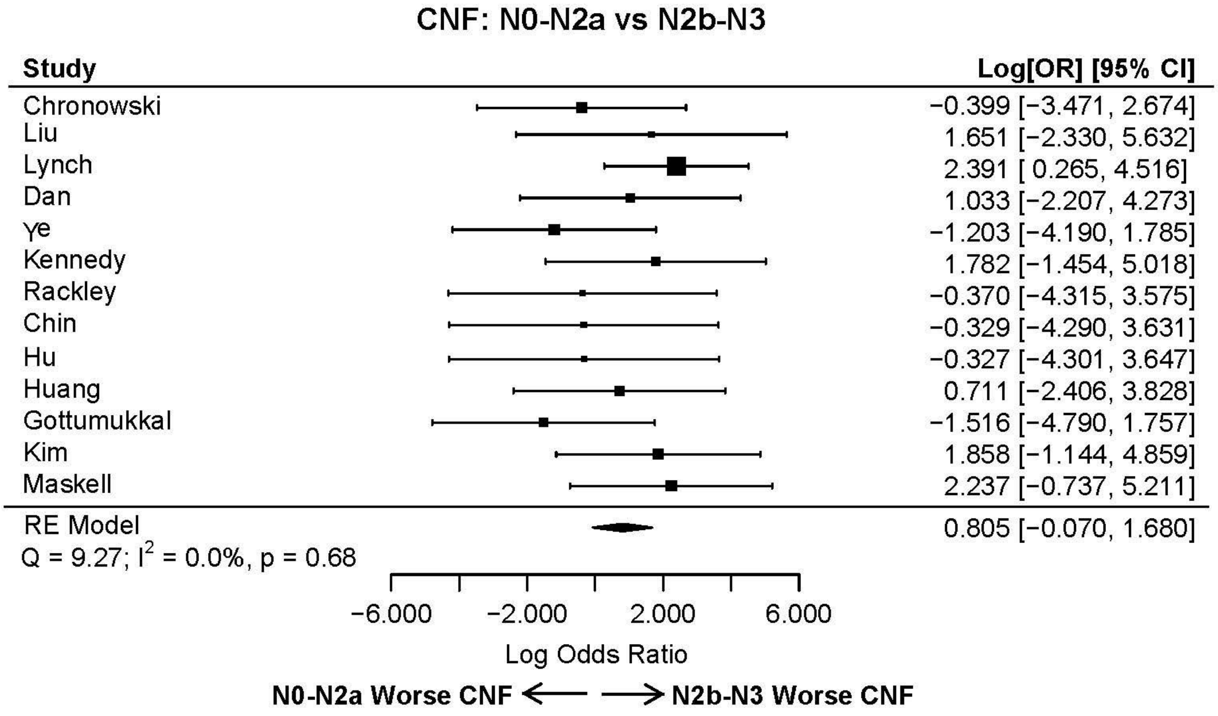

© 2023 Razavian NB et al. *JAMA Network Open*.

eFigure 10. Contralateral Neck Failure Rates Grouped by RT Type

Pooled contralateral neck failure outcomes are shown grouped by studies that reported contralateral neck failure outcomes from patients treated with ipsilateral or bilateral RT

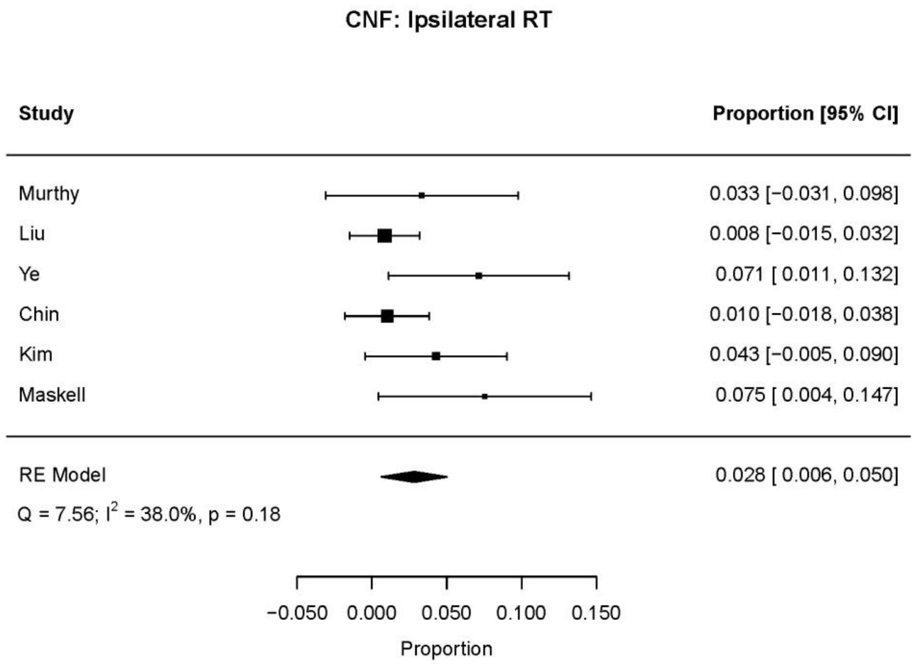

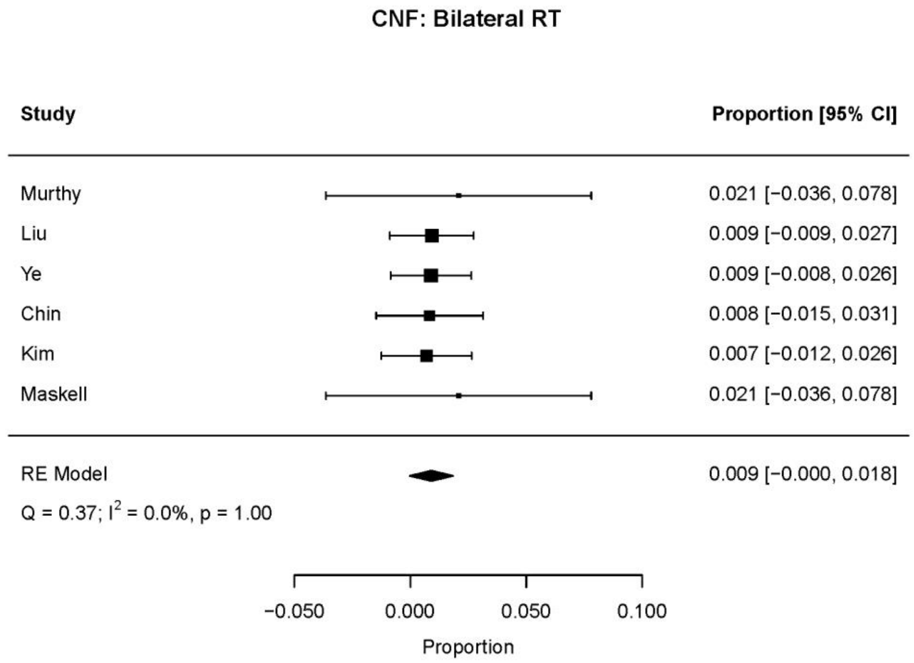

© 2023 Razavian NB et al. *JAMA Network Open*.

eFigure 11. Association Between Treatment and Risk Factors With Contralateral Neck Failure

Meta-regression analyses were performed to assess for association between contralateral neck failure (CNF) rate and proportion of patients with/receiving (A) intensity modulated radiation therapy (IMRT), (B) neck dissection, (C) chemotherapy, (D) smoking history, and (E) HPV+ disease. Association between CNF and (F) median follow-up time, (G) male sex, and (H) median age were also assessed. Regression coefficients (β) and P-values from each mixed-effects model are displayed.

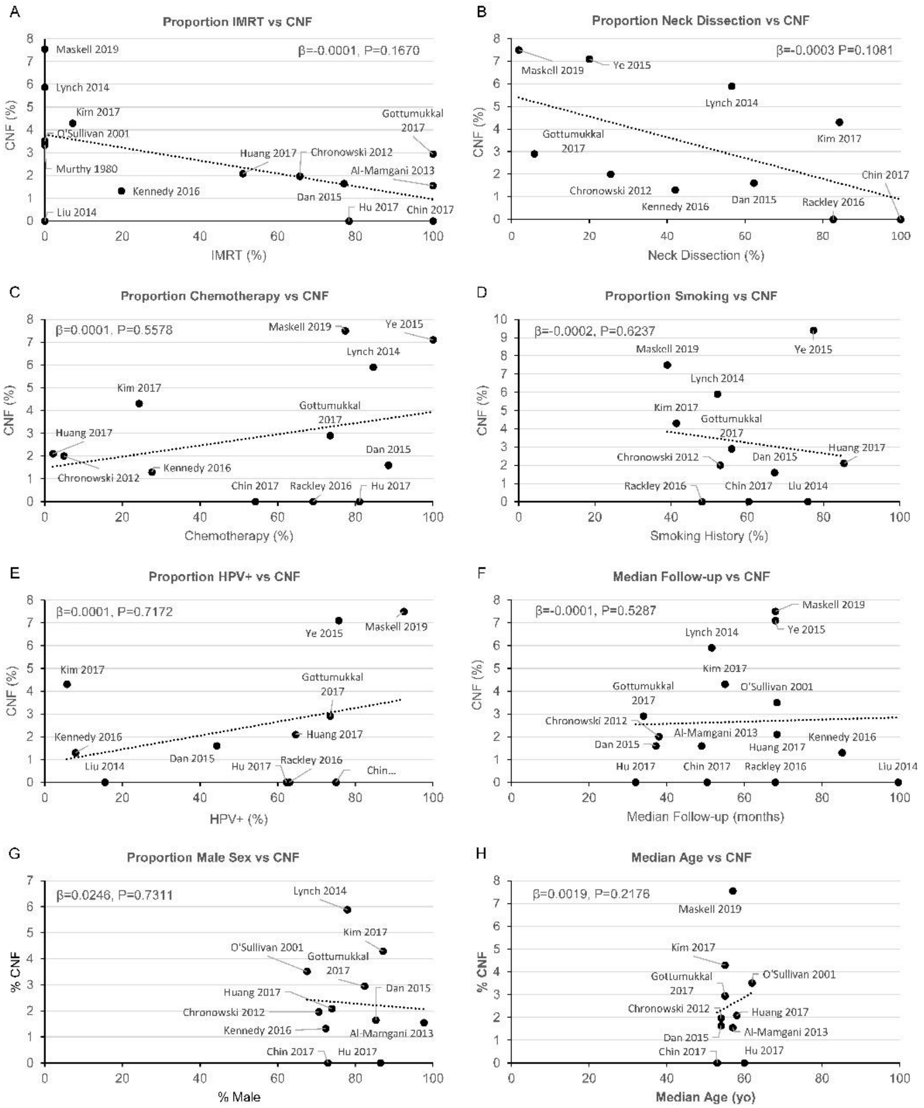

© 2023 Razavian NB et al. *JAMA Network Open*.

eFigure 12. Heterogeneity After Excluding Outlier Feeding Tube Studies

In cases of high heterogeneity (e.g. I2 >50%) sensitivity analysis was performed to identify causes of heterogeneity. Here, Cook's distance method was used to identify outlier studies. Among the feeding tube studies, only 1 outlier (Gottumukkal et al) was identified. After removal of this outlier study, pooled rate of feeding tube use was similar (11.6% from 13.3%), but heterogeneity remained high (I^2 =61.5% from 71.3%).

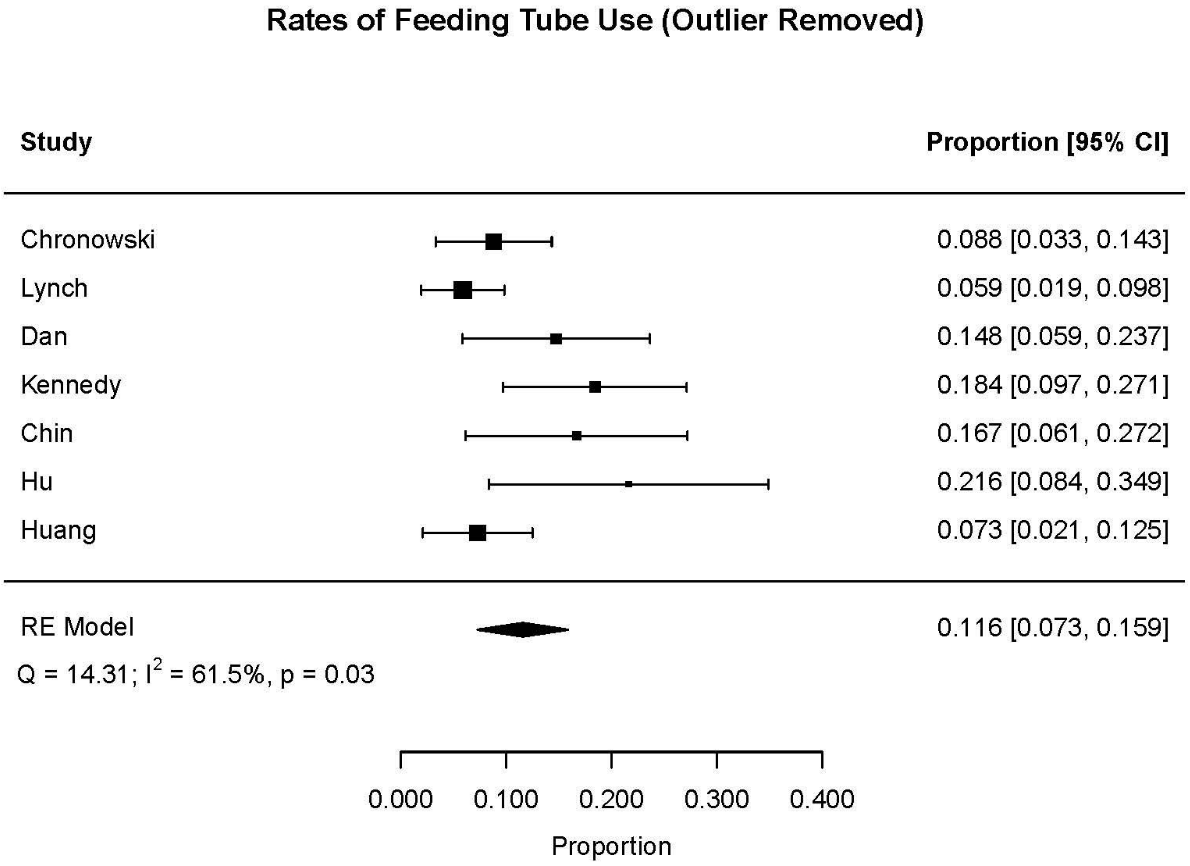

© 2023 Razavian NB et al. *JAMA Network Open*.

eTable 1. Treatment Characteristics of Studies Included for Meta-analysis

Study	Pub Year	Design	Country	Median Age (range)	Median Follow-up (range)	Median Dose (range)	IMRT (%)	Chemo (%)	Neck Dissection (%)	CNF End Point Assessment
Murthy	1980	RR	USA	-	60 mo (45-60)	-	-	-	-	PA
Jackson	1999	RR	CAN	-	60 mo (50-66)	-	-	3.4	-	PA
O'Sullivan	2001	RR	CAN	62 yo (N/A)	68.4 mo (N/A)	50 Gy (N/A)	0		-	NA
Chronowski	2012	RR	USA	54 yo (38-72)	38 mo (N/A)	66 Gy (4.4-72)	65.7	4.9	25.5	PA
Al-Mamgani	2013	RR	NL	57 yo (37-85)	49 mo (N/A)	N/A (58-70)	100	-	-	CA, RA, PA
Liu	2014	RR	AUS	58 yo (36-89)	99.6 mo (N/A)	N/A (66-70)	0	-	-	NA
Lynch	2014	RR	UK	51.6 yo (N/A)	N/A (46.75-65)	-	84.6	100	-	PA
Dan	2015	RR	USA	54 yo (38-72)	37.2 mo (N/A)	N/A (54-70)	77.1	88.5	62.3	PA, RA
Ye	2015	RR	CAN	68 yo (N/A)	N/A (50-70)	-	100	21.4	-	PA
Kennedy	2016	RR	USA	85.2 yo (N/A)	74.4 mo (50-76.8)	19.7 Gy (N/A)	27.6	42.1	-	CA, RA, PA
Rackley	2016	RR	UK	55 yo (35-82)	68 mo (N/A)	N/A (50-66)	-	69.1	82.7	CA
Chin	2017	RR	USA	53 yo (27-84)	50.4 mo (N/A)	N/A (52-66)	100	54.2	100	CA, RA
Hu	2017	P	USA	60 yo (42-74)	32 mo (N/A)	66 Gy (50-70)	78.4	81.1	-	CA, RA
Huang	2017	RR	CAN	58 yo (42-93)	68.4 mo (N/A)	N/A (51-70)	51	2.1	-	CA, RA, PA
Gottumukkal	2017	RR	USA	55 yo (40-68)	34 mo (N/A)	67 Gy (N/A)	100	73.5	5.9	PA
Kim	2017	RR	KR	55 yo (31-76)	55 mo (N/A)	N/A (36-70)	7.1	24.3	84.3	PA
Maskell	2019	RR	UK	57 yo (36-89)	68 mo (N/A)	N/A (36-55)	0	77.4	1.9	PA

Abbreviations: retrospective review (RR), prospective study (P), intensity modulated radiation therapy (IMRT), contralateral neck failure (CNF), pathologic assessment (PA), narrative assessment (NA), radiographic assessment (RA), clinical assessment (CA)

© 2023 Razavian NB et al. *JAMA Network Open*.

eTable 2. Baseline Characteristics of Studies Included for Meta-analysis

Study	Degree of Extension	Staging Edition	Staging Imaging Used?	HPV+ (%)	Smoking (%)	Male (%)
Murthy	Not Provided	Prior to AJCC 7th	No Imaging Used	-	-	-
Jackson	Extension Allowed	Prior to AJCC 7th	No Imaging Used	-	-	-
O'Sullivan	Not Provided	Prior to AJCC 7th	No Imaging Used	-	-	67.5
Chronowski	Extension Allowed	AJCC 7th	No Imaging Used	-	52.9	70.6
Al-Mamgani	Extension Allowed	Not Provided	No Imaging Used	-	-	68.1
Liu	Extension Allowed	Prior to AJCC 7th	Imaging	15.5	75.9	76.1
Lynch	No Extension Allowed	AJCC 7th	No Imaging Used	-	52.2	77.9
Dan	No Extension Allowed	Not Provided	Imaging	44.3	67.2	85.2
Ye	Not Provided	Not Provided	Used Imaging	75.7	77.4	76.9
Kennedy	No Extension Allowed	AJCC 7th	No Imaging Used	7.9	-	72.3
Rackley	Extension Allowed	Not Provided	Used Imaging	63	48.1	72.8
Chin	Extension Allowed	AJCC 7th	Used Imaging	75	60.4	72.9
Hu	Extension Allowed	Not Provided	No Imaging Used	62.2	-	86.5
Huang	Extension Allowed	AJCC 7th	Used Imaging	64.6	85.4	74.0
Gottumukkal	Extension Allowed	AJCC 7th	Used Imaging	73.5	55.9	82.4
Kim	Not Provided	Not Provided	Used Imaging	5.7	41.4	87.1
Maskell	Extension Allowed	AJCC 7th	Used Imaging	92.5	39	-

© 2023 Razavian NB et al. *JAMA Network Open*.

eTable 3. T and N Stages of Studies Included for Meta-analysis

Study	T1	T2	T3	T4	N0	N1	N2a	N2b	N3
Murthy	-	-	-	-	14	6	-	-	2
Jackson	-	-	-	-	101	54	-	-	16
O'Sullivan	73	118	30	7	133	56	-	-	3
Chronowski	52	33	-	-	33	23	21	22	-
Al-Mamgani	-	-	-	-	-	-	-	-	-
Liu	-	-	-	-	25	14	10	4	5
Lynch	57	74	5	-	28	20	31	55	2
Dan	29	30	2	-		15	14	31	1
Ye	22	28	3	-	15	18	7	11	2
Kennedy	41	35	-	-	27	15	8	26	-
Rackley	40	41	-	-	18	3	12	48	-
Chin	23	18	6	0	5	5	10	28	0
Hu	11	22	4		3	9	3	21	-
Huang	40	56	-	-	52	25	11	8	-
Gottumukkal	20	11	2	1		6	8	19	1
Kim	23	47	-	-	10	16	6	38	-
Maskell	27	24	2	-	10	10	5	28	-

© 2023 Razavian NB et al. *JAMA Network Open*.

eTable 4. Contralateral Neck Failure (CNF) Outcomes of Included Studies by RT Type

Study	Ipsilateral RT	Ipsilateral RT - CNF	Bilateral RT	Bilateral RT - CNF
Murthy	30	1	23	0
Jackson	178	4	-	-
O'Sullivan	228	8	-	-
Chronowski	102	2	-	-
Al-Mamgani	129	2	-	-
Liu	58	0	108	1
Lynch	136	8	-	-
Dan	61	1	-	-
Ye	70	5	112	1
Kennedy	76	1	-	-
Rackley	81	0	-	-
Chin	48	0	59	0
Hu	37	0	-	-
Huang	96	2	-	-
Gottumukkal	34	1	-	-
Kim	70	3	70	0
Maskell	53	4	23	0

© 2023 Razavian NB et al. *JAMA Network Open*.

eTable 5. Contralateral Neck Failure (CNF) Following Ipsilateral RT by T and N Stages

Study	T1 CNF	T2 CNF	T3 CNF	T4 CNF	N0 CNF	N1 CNF	N2a CNF	N2b CNF	N3 CNF
Murthy	-	-	-	-	0	0	-	-	0
Jackson	-	-	-	-	2	2	-	-	0
O'Sullivan	0	4	3	1	0	8	-	-	0
Chronowski	0	1	-	-	2	0	0	0	-
Al-Mamgani	-	-	-	-	-	-	-	-	-
Liu	-	-	-	-	0	0	0	0	0
Lynch	0	7	1	-	0	1	0	7	0
Dan	0	1	0	-	-	0	0	1	0
Ye	1	2	1	-	0	3	1	0	0
Kennedy	1	0	-	-	0	0	0	1	-
Rackley	0	0	-	-	0	0	0	0	-
Chin	0	0	0	0	0	0	0	0	0
Hu	0	0	0		0	0	0	0	-
Huang	0	2	-	-	0	2	0	0	-
Gottumukkal	0	1	0	0		1	0	0	0
Kim	0	3	-	-	0	0	0	3	-
Maskell	4	0	0	0	0	0	0	4	-

© 2023 Razavian NB et al. *JAMA Network Open*.

eTable 6. Toxicity Outcomes—Grade 3 or Greater Xerostomia and G Tube Use

Study	Xerostomia Assessed	Xerostomia Developed	G Tube Assessed	G Tube Required
Murthy	-	-	-	-
Jackson	-	-	-	-
O'Sullivan	-	-	-	-
Chronowski	-	-	102	9
Al-Mamgani	-	-	-	-
Liu	-	-	-	-
Lynch	-	-	136	8
Dan	61	0	9	61
Ye	-	-	-	-
Kennedy	-	-	76	14
Rackley	-	-	-	-
Chin	40	1	48	8
Hu	37	0	37	8
Huang	96	0	96	7
Gottumukkal	-	-	32	10
Kim	70	1	-	-
Maskell	-	-	-	-

© 2023 Razavian NB et al. *JAMA Network Open*.

eTable 7. Quality of Included Studies per MINORS Criteria

Author	Study Type	Clearly stated aim	Inclusion of consecutive patients	Prospective data collection	Endpoints appropriate of study	Unbiased assessment of study endpoint	Follow-up period appropriate to study aim	Loss to follow up does not exceed the proportion experiencing the major endpoint	Total score	CNF Assessment	Toxicity Assessment
Murthy	RR	2	2	N/A	1	1	0	1	7	PA	Not Reported
Jackson	RR	2	2	N/A	1	1	0	1	7	PA	Narrative, limited
O'Sullivan	RR	2	2	N/A	1	1	2	2	10	NA	Narrative, limited
Chronowski	RR	2	2	N/A	1	1	2	2	10	PA	Narrative, limited
Al-Mamgani	RR	2	2	N/A	2	1	2	2	11	CA, RA, PA	CTCAE
Liu	RR	2	2	N/A	1	1	2	2	10	NA	Graded, scale not provided
Lynch	RR	2	2	N/A	1	1	2	2	10	PA	CTCAE, RTOG
Dan	RR	2	2	N/A	2	1	2	2	11	PA, RA	CTCAE, RTOG
Ye	RR	2	2	N/A	1	1	2	2	10	PA	Not Reported
Kennedy	RR	2	2	N/A	2	1	2	2	11	CA, RA, PA	CTCAE
Rackley	RR	2	2	N/A	2	1	2	2	11	CA	CTCAE, RTOG
Chin	RR	2	2	N/A	2	1	2	2	11	CA, RA	CTCAE, MDADI, XQ
Hu	P	2	2	2	2	1	2	2	13	CA, RA	RTOG, NCCN Distress Thermometer
Huang	RR	2	2	N/A	2	1	2	2	11	CA, RA, PA	Graded, scale not provided
Gottumukkal	RR	2	2	N/A	1	1	2	2	10	PA	Not Reported
Kim	RR	2	2	N/A	1	1	2	2	10	PA	RTOG
Maskell	RR	2	2	N/A	1	1	2	2	10	PA	Narrative, limited

Abbreviations: contralateral neck failure (CNF), retrospective review (RR), prospective study (P), pathologic assessment (PA), narrative assessment (NA), radiographic assessment (RA), common terminology criteria for adverse events (CTCAE), radiation therapy oncology group (RTOG), national comprehensive cancer network (NCCN), MD Anderson dysphagia inventory (MDADI), xerostomia questionnaire (XQ)

© 2023 Razavian NB et al. *JAMA Network Open*.

eTable 8. Definition of N2b Disease by Staging Edition

	Staging Edition				
	**AJCC 1st ed**	**AJCC 3rd ed**	**AJCC 4th ed**	**AJCC 5th ed**	**AJCC 7th ed**
**N2b**	Multiple, clinically positive homolateral nodes, none >6cm	Metastasis in multiple ipsilateral lymph nodes, none more than 6 cm in greatest dimension	Metastasis in multiple ipsilateral lymph nodes, none more than 6 cm in greatest dimension	Metastasis in multiple ipsilateral lymph nodes, none more than 6 cm in greatest dimension	Metastasis in multiple ipsilateral lymph nodes, none more than 6 cm in greatest dimension

Abbreviations: American Joint Committee on Cancer (AJCC)

© 2023 Razavian NB et al. *JAMA Network Open*.

eReferences

1. Rackley TP, Namelo WC, Palaniappan N, Cole N, Owens DM, Evans M. Unilateral radiotherapy for surgically resected lateralized squamous cell carcinoma of the tonsil. *Head Neck*. 2017;39(1):17-23.
2. Chronowski GM, Garden AS, Morrison WH, et al. Unilateral radiotherapy for the treatment of tonsil cancer. *Int J Radiat Oncol Biol Phys*. 2012;83(1):204-209.
3. Hu KS, Mourad WF, Gamez M, et al. Low rates of contralateral neck failure in unilaterally treated oropharyngeal squamous cell carcinoma with prospectively defined criteria of lateralization. *Head Neck*. 2017;39(8):1647-1654.
4. Al-Mamgani A, van Rooij P, Fransen D, Levendag P. Unilateral neck irradiation for well-lateralized oropharyngeal cancer. *Radiother Oncol*. 2013;106(1):69-73.
5. Chin RI, Rao YJ, Hwang MY, et al. Comparison of unilateral versus bilateral intensity-modulated radiotherapy for surgically treated squamous cell carcinoma of the palatine tonsil. *Cancer*. 2017;123(23):4594-4607.
6. Dan TD, Raben D, Schneider CJ, et al. Freedom from local and regional failure of contralateral neck with ipsilateral neck radiotherapy for node-positive tonsil cancer: updated results of an institutional clinical management approach. *Oral Oncol*. 2015;51(6):616-621.
7. Gottumukkala S, Pham NL, Sumer B, et al. Risk of contralateral nodal failure following ipsilateral IMRT for node-positive tonsillar cancer. *Oral Oncol*. 2017;75:35-38.
8. Huang SH, Waldron J, Bratman SV, et al. Re-evaluation of ipsilateral radiation for T1-T2N0-N2b tonsil carcinoma at the Princess Margaret Hospital in the human papillomavirus era, 25 years later. *Int J Radiat Oncol Biol Phys*. 2017;98(1):159-169.
9. Jackson SM, Hay JH, Flores AD, et al. Cancer of the tonsil: the results of ipsilateral radiation treatment. *Radiother Oncol*. 1999;51(2):123-128.
10. Kennedy WR, Herman MP, Deraniyagala RL, et al. Ipsilateral radiotherapy for squamous cell carcinoma of the tonsil. *Eur Arch Otorhinolaryngol*. 2016;273(8):2151-2156.
11. Kim Y, Cho KH, Moon SH, et al. Comparison of the clinical outcomes of patients with squamous cell carcinoma of the tonsil

© 2023 Razavian NB et al. *JAMA Network Open*.

receiving postoperative ipsilateral versus bilateral neck radiotherapy: a propensity score matching analysis (KROG 11-07). *Cancer Res Treat*. 2017;49(4):1097-1105.
12. Liu C, Dutu G, Peters LJ, Rischin D, Corry J. Tonsillar cancer: the Peter MacCallum experience with unilateral and bilateral irradiation. *Head Neck*. 2014;36(3):317-322.
13. Lynch J, Lal P, Schick U, et al. Multiple cervical lymph node involvement and extra-capsular extension predict for contralateral nodal recurrence after ipsilateral radiotherapy for squamous cell carcinoma of the tonsil. *Oral Oncol*. 2014;50(9):901-906.
14. Maskell D, Buckley H, Sission K, Roques T, Geropantas K. Ipsilateral neck radiotherapy in N2b well-lateralized tonsil cancer—approach with caution. *Head Neck*. 2019;41(9):2937-2946.
15. Murthy AK, Hendrickson FR. Is contralateral neck treatment necessary in early carcinoma of the tonsil? *Int J Radiat Oncol Biol Phys*. 1980;6(1):91-94.
16. O'Sullivan B, Warde P, Grice B, et al. The benefits and pitfalls of ipsilateral radiotherapy in carcinoma of the tonsillar region. *Int J Radiat Oncol Biol Phys*. 2001;51(2):332-343.
17. Ye A, Bradley KL, Kader H, Wu J, Hay JH. Patterns of relapse in squamous cell carcinoma of the tonsil—unilateral vs bilateral radiation in the HPV-era. *Cureus*. 2015;7(9):e322.

© 2023 Razavian NB et al. *JAMA Network Open*.
