# Supplementary material for: Association of Unilateral Radiotherapy With Contralateral Lymph Node Failure Among Patients With Squamous Cell Carcinoma of the Tonsil: A Systematic Review and Meta-analysis
Source: JAMA Netw Open. 2023 Feb 8;6(2):e2255209. doi: 10.1001/jamanetworkopen.2022.55209 (PMC9909500; doi:10.1001/jamanetworkopen.2022.55209)
Supplement: Supplement 2. — Data Sharing Statement [file jamanetwopen-e2255209-s002.pdf]

Data Sharing Statement

Razavian. Association of Unilateral Radiotherapy With Contralateral Lymph Node Failure Among Patients With Squamous Cell Carcinoma of the Tonsil. *JAMA Netw Open*. Published February 08, 2023. doi:10.1001/jamanetworkopen.2022.55209

Data
Data available: Yes
Data types: Deidentified participant data
How to access data: ryhughes@wakehealth.edu
When available: With publication

Supporting Documents
Document types: None

Additional Information
Who can access the data: anyone requesting the data
Types of analyses: for any purpose
Mechanisms of data availability: upon formal request to the corresponding author
